# Personalized Vascularized Models of Breast Cancer Desmoplasia Reveal Biomechanical Determinants of Drug Delivery to the Tumor

**DOI:** 10.1002/advs.202402757

**Published:** 2024-07-23

**Authors:** Giovanni S. Offeddu, Elena Cambria, Sarah E. Shelton, Kristina Haase, Zhengpeng Wan, Luca Possenti, Huu Tuan Nguyen, Mark R. Gillrie, Dean Hickman, Charles G. Knutson, Roger D. Kamm

**Affiliations:** ^1^ Department of Biological Engineering Massachusetts Institute of Technology Cambridge MA 02139 USA; ^2^ European Molecular Biology Laboratory European Molecular Biology Laboratory Barcelona Barcelona 08003 Spain; ^3^ LaBS Department of Chemistry Materials and Chemical Engineering Politecnico di Milano Milan 20133 Italy; ^4^ Data Science Unit Fondazione IRCCS Istituto Nazionale dei Tumori Milan 20133 Italy; ^5^ Department of Medicine Snyder Institute for Chronic Diseases University of Calgary Calgary Alberta T2N 2T9 Canada; ^6^ Amgen Research Amgen Inc. 360 Binney Street Cambridge MA 02142 USA

**Keywords:** hyaluronic acid, microphysiological systems, permeability, TGFβ mechanotherapy, tumor vascularization, vascular glycocalyx

## Abstract

Desmoplasia in breast cancer leads to heterogeneity in physical properties of the tissue, resulting in disparities in drug delivery and treatment efficacy among patients, thus contributing to high disease mortality. Personalized in vitro breast cancer models hold great promise for high‐throughput testing of therapeutic strategies to normalize the aberrant microenvironment in a patient‐specific manner. Here, tumoroids assembled from breast cancer cell lines (MCF7, SKBR3, and MDA‐MB‐468) and patient‐derived breast tumor cells (TCs) cultured in microphysiological systems including perfusable microvasculature reproduce key aspects of stromal and vascular dysfunction causing impaired drug delivery. Models containing SKBR3 and MDA‐MB‐468 tumoroids show higher stromal hyaluronic acid (HA) deposition, vascular permeability, interstitial fluid pressure (IFP), and degradation of vascular HA relative to models containing MCF7 tumoroids or models without tumoroids. Interleukin 8 (IL8) secretion is found responsible for vascular dysfunction and loss of vascular HA. Interventions targeting IL8 or stromal HA normalize vascular permeability, perfusion, and IFP, and ultimately enhance drug delivery and TC death in response to perfusion with trastuzumab and cetuximab. Similar responses are observed in patient‐derived models. These microphysiological systems can thus be personalized by using patient‐derived cells and can be applied to discover new molecular therapies for the normalization of the tumor microenvironment.

## Graphical abstract

Patient‐specific breast cancer models incorporate functional human microvasculature that can be perfused with therapeutic molecules to test their efficacy in normalizing the desmoplastic tumor microenvironment to healthy tissue levels and to promote effective drug delivery. These pathophysiologically relevant models can be used to discover new therapeutic targets and to inform clinical strategies.

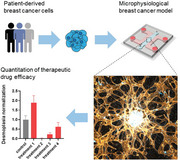

## 1 Introduction

Desmoplasia is a key process underlying disease progression in breast cancer. The induced physical heterogeneity in the breast tissue presents a major obstacle to treatment.^[^ ^1^, ^2^ ^]^ Breast TCs are largely responsible for the desmoplastic remodeling of the tumor microenvironment, both directly through aberrant deposition of extracellular matrix (ECM) proteins and indirectly through activation of stromal cells.^[^ ^3^, ^4^, ^5^, ^6^ ^]^ Increased ECM density and reduced vascular perfusion, vascular barrier function, and lymphatic drainage all contribute to increased IFP in the breast tumor microenvironment, resulting in impaired drug delivery from the blood to the TCs.^[^ ^6^, ^7^, ^8^, ^9^ ^]^ Therapeutic strategies to normalize the desmoplastic breast cancer microenvironment are currently in clinical trials.^[^ ^10^ ^]^ However, the patient‐to‐patient heterogeneity of breast cancer, and related variable response to treatments, make it critical to identify personalized strategies with the greatest therapeutic potential.

Patient‐derived TCs assembled into structures with increasing complexity and pathophysiological relevance, including single‐cell‐type spheroids,^[^ ^11^ ^]^ multi‐cell‐type tumoroids,^[^ ^12^, ^13^ ^]^ and stem cell‐derived organoids,^[^ ^14^ ^]^ have recently attracted attention, as they can be used to rapidly evaluate the response to molecular therapies.^[^ ^15^ ^]^ Incorporating these structures into microphysiological models presents the opportunity to broaden their scope and capture key morphological and functional aspects of the tumor microenvironment.^[^ ^16^, ^17^, ^18^, ^19^ ^]^ An additional attractive feature of microfluidic‐based models is the extremely fine control over the mechanical and biochemical stimuli imparted on the cells within.^[^ ^20^, ^21^ ^]^ However, current models have so far been unsuccessful in recapitulating the complexity of the desmoplastic breast cancer microenvironment,^[^ ^22^ ^]^ particularly the aberrant ECM and vasculature, which jointly determine drug delivery to the tumor. Harnessing the potential of microphysiological models to recapitulate the pathological complexity of the aberrant tumor microenvironment in breast cancer desmoplasia may offer the possibility to discover new therapeutic strategies, thereby improving clinical care for breast cancer patients.

We have recently developed microphysiological models including cancer spheroids assembled from ovarian and lung cancer cell lines and human microvascular networks (MVNs).^[^ ^23^ ^]^ These MVNs can be perfused with therapeutic molecules to assess their permeability across the vascular endothelium and the resulting TC death. In this work, we expand on this methodology to culture tumoroids containing patient‐derived breast TCs or breast cancer cell lines and evaluate their microenvironments to demonstrate differential desmoplastic stromal and vascular remodeling, as well as responsiveness to interventions that normalize the tumor microenvironment. We further show that these vascularized tumor models can provide quantitative metrics to test different therapeutic strategies and empower the discovery of new molecular targets to normalize the desmoplastic breast cancer microenvironment.

## 2 Results

### 2.1 Vascularized Tumoroids‐On‐Chip Differentially Remodel Their Stroma through Increased Production of Hyaluronic Acid

**Figure 1:**
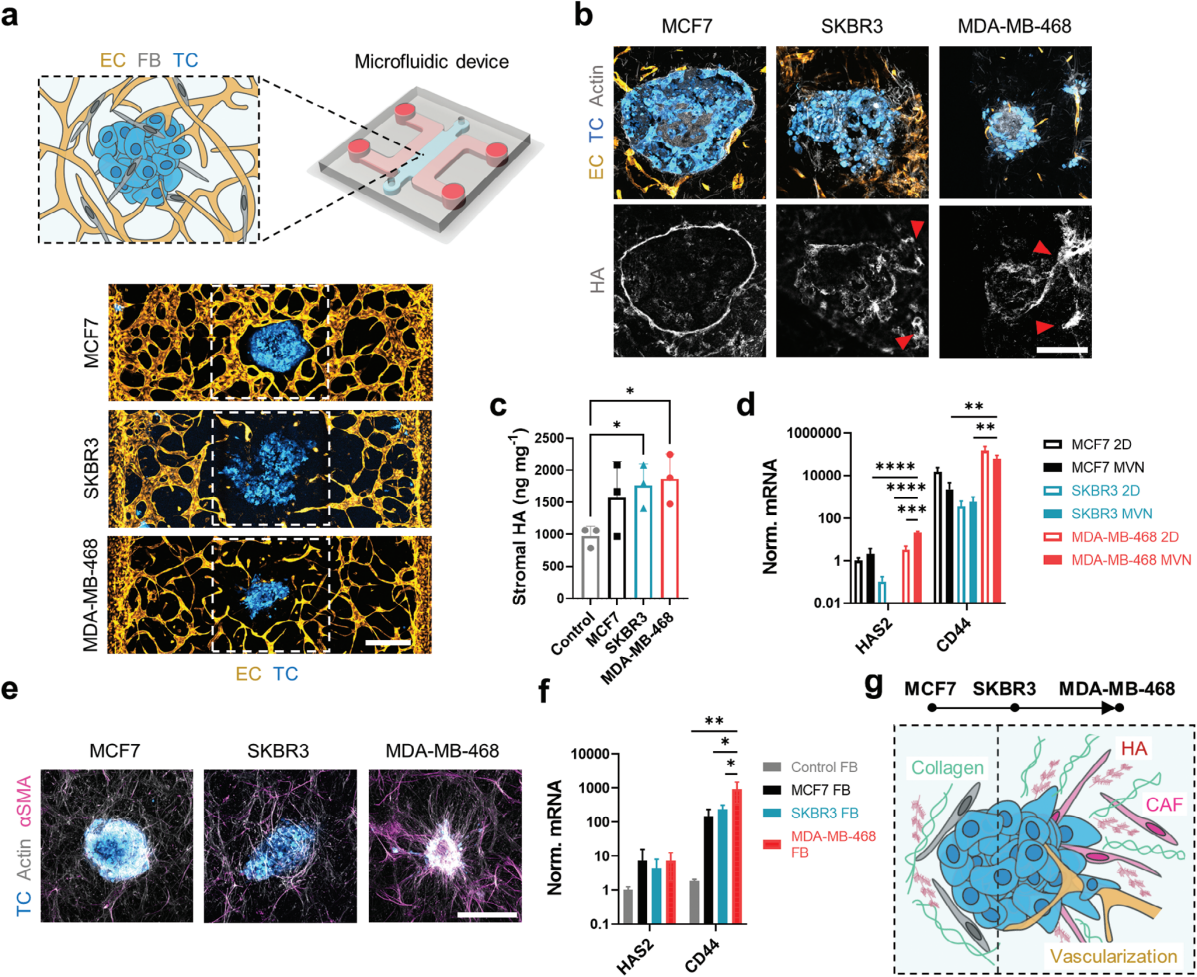
Tumoroids assembled from breast cancer cell lines differentially remodel their surrounding stroma. a) Schematic diagram (top) and projected confocal microscopy images (bottom) of tumoroids in the MVN devices. b) Projected confocal images of tumoroid cryosections showing HA localization. The red arrows indicate HA traces left by SKBR3 and MDA‐MB‐468 cells migrating out of the tumoroids. The scale bar is 200 µm. c) Quantification of stromal HA concentration in 1 mm biopsies centered at the tumoroids in the MVN devices. d) Expression of HA‐associated genes in TCs cultured in 2D or collected from MVN devices; *n* = 3. The missing bar in this figure indicates a nondetectable *HAS2* gene expression in SKBR3 MVNs. e) Projected confocal images of FBs expressing αSMA and becoming cancer‐associated FBs (CAFs) in the tumoroid microenvironments. The scale bar is 500 µm. f) Expression of HA‐associated genes in FBs collected from MVN devices; *n* = 3. g) Tumoroid microenvironments and their representation of the progression of breast cancer desmoplasia: from MCF7, where HA and collagen form a dense layer around the tumoroids, to MDA‐MB‐468, where HA and collagen expression increases together with the presence of CAFs and tumoroid vascularization. Significance assessed by one‐way ANOVA after confirming normal distribution of the data; in (d), significance is shown only between 2D and MVNs for each TC type, and between TC types in MVNs; *p* < 0.05 *, *p* < 0.01 **, *p* < 0.001 ***, *p* < 0.0001 ****.

We first used breast cancer cell lines representative of three main disease molecular sub‐types^[^ ^24^ ^]^ to characterize the model: MCF7, SKBR3, and MDA‐MB‐468. MCF7 cells are estrogen receptor (ER)+, progesterone receptor (PR)+, human epidermal growth factor receptor 2 (HER2/ERBB2)‐, and epidermal growth factor receptor (EGFR)‐. SKBR3 cells are ER‐/PR‐/HER2+/EGFR‐. MDA‐MB‐468 cells are ER‐/PR‐/HER2‐/EGFR+. Tumoroids with a diameter of approximately 500 µm were formed by coculturing TCs with human fibroblasts (FBs) in non‐adherent well plates for 4 d, which allowed the formation of stable cell aggregates even for TCs that would normally not aggregate in monoculture (SKBR3 and MDA‐MB‐468). We then cocultured the tumoroids in 3D gels within microfluidic devices containing human MVNs (**Figure** **1**a) as recently described.^[^ ^23^ ^]^ MVNs self‐assembled over 7 d from human endothelial cells (ECs) and the same FBs used to form the tumoroids. Differential gene expression of target receptors was confirmed for the different TC types (Figure S1a, Supporting Information) and was shown to increase in the MVN devices compared to 2D culture (Figure S1b, Supporting Information), leading to measurable levels of proteins in the microenvironment of all three tumoroid types (Figure S1c, Supporting Information). Expression of receptors was co‐localized with the TCs (Figure S1d, Supporting Information), and sectioning of tumoroids in MVN devices revealed a cytokeratin‐rich core of dead cells, where receptor expression was lost (Figures S1d and S2a, Supporting Information). The tumoroid dead core may be the result of hypoxia‐induced necrosis, as previously observed in tumors in vivo and TC aggregates larger than 500 µm,[^24^] and as suggested by high levels of HIF‐1α expression in the tumoroids (Figure S2b, Supporting Information). These observations cumulatively show that enhanced pathophysiological receptor expression and tumor architecture can be recapitulated in the vascularized tumoroid models.

The pathophysiological relevance of the vascularized tumoroid models extended to the remodeling of the microenvironment by the TCs. MCF7 cells remained tightly packed in the tumoroids, consistently with their high expression of the epithelial junction marker E‐cadherin (Figure S2c, Supporting Information), while the more mesenchymal SKBR3 and MDA‐MB‐468 cells migrated out of the tumoroids to invade the stroma (Figure 1a,b). Increased HA concentration is a hallmark of breast cancer desmoplasia and is associated with poor prognosis.^[^ ^25^ ^]^ HA deposition at the periphery of the tumoroids was higher compared to the surrounding microenvironment (Figure 1b). Yet, HA localization varied between tumoroids: HA formed a dense layer around MCF7 tumoroids, whereas it became progressively more diffuse and eventually localized with migrating TCs in SKBR3 and MDA‐MB‐468 tumoroids (Figure 1b).

We sought to understand whether the increased HA concentration measured in the tumoroid microenvironments (Figure 1c) was a result of TC production. Gene expression analysis revealed higher expression of *hyaluronic acid synthase 2 (HAS2)*, which produces high molecular weight HA,^[^ ^26^ ^]^ and HA receptor *CD44* by MDA‐MB‐468 cells compared to the other TCs (Figure 1d). CD44 is an important adhesion molecule used by TCs to invade the stroma,^[^ ^25^ ^]^ and its higher expression in MDA‐MB‐468 cells is consistent with their enhanced migratory phenotype. Interestingly, *HAS2* expression in MDA‐MB‐468 cells was significantly higher in the MVN devices compared to 2D monoculture (Figure 1d). Conversely, SKBR3 cells showed the lowest expression of *HAS2* and *CD44* among the cell lines (Figure 1d) and increased expression for HA‐degrading hyaluronidase 1 (*HYAL1*, Figure S3a, Supporting Information), suggesting lower HA deposition by those TCs and the presence of an alternative source of HA in their microenvironment.

Through an altered fibrotic phenotype that develops as a result of signaling in the tumor microenvironment, cancer‐associated FBs (CAFs) produce aberrant amounts of HA and collagen I that promote increased TC migration and tumor proliferation.^[^ ^5^ ^]^ Immunofluorescence analysis of a key marker of CAFs,^[^ ^27^ ^]^ alpha smooth muscle actin (αSMA), revealed an increasing transformation of FBs into CAFs, especially in the SKBR3, and MDA‐MB‐468 tumoroid microenvironments (Figure 1e). We also noted a correlation between the abundance of CAFs and expression of fibroblast growth factor (FGF) in the SKBR3 and MDA‐MB‐468 tumoroids (Figure S2c, Supporting Information).^[^ ^28^ ^]^ We sought to understand the role of FBs in HA production within the MVN devices, and found increased expression of *CD44* (Figure 1f) and hyaluronidase 2 (*HYAL2*, Figure S3b, Supporting Information), a marker of HA metabolism,^[^ ^29^ ^]^ in FBs in the vicinity (1 mm) of the tumoroids compared to control FBs in MVN devices without tumoroids. FBs appeared more densely associated with the MDA‐MB‐468 tumoroids, for which we observed increased collagen I deposition (Figure S3c, Supporting Information). Remarkably, collagen I aligned radially to the MDA‐MB‐468 tumoroids (Figure S3c, Supporting Information), as seen in the progression of breast cancer desmoplasia, where TCs form and follow migration tracks to invade the stroma.^[^ ^4^ ^]^

Overall, these results show that different tumoroid types can alter their microenvironment in drastically different ways. In addition, despite the relative simplicity of cancer cell lines compared to patient‐derived TCs, the tumoroids are capable of building complex microenvironments in the MVN devices that mimic key aspects of the progression of breast cancer desmoplasia.^[^ ^4^ ^]^ Specifically, the MCF7 tumoroids appear to be representative of an early disease stage, where TCs still possess a primarily epithelial phenotype and a fibrotic ECM layer forms around the tumoroids and restricts TC migration and invasion (Figure 1g). The MDA‐MB‐468 tumoroids, instead, may be representative of a later disease stage, whereby TCs aggressively invade the tumoroid microenvironment through a denser ECM deposited by both TCs and CAFs (Figure 1g). SKBR3 tumoroids lie somewhat in between these two extremes (Figure 1g). MDA‐MB‐468 tumoroids also recapitulated an additional feature of disease progression in that they often became vascularized by the surrounding MVNs (Figure S4, Supporting Information). We next assessed whether these vascular and ECM changes resulted in differences in drug delivery to the tumoroids.

### 2.2 Loss of Vascular Hyaluronic Acid Contributes to Increased Vascular Permeability and Interstitial Fluid Pressure in the Tumoroids

**Figure 2:**
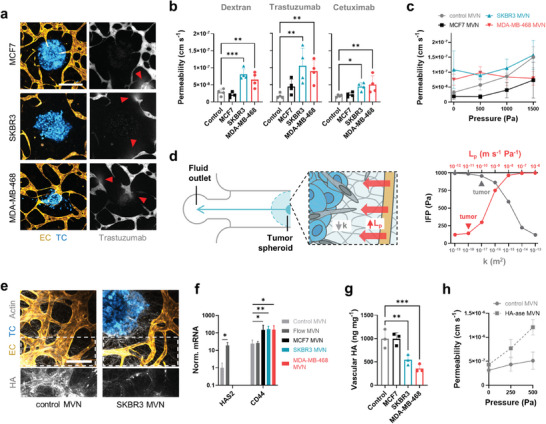
Increased vascular permeability and interstitial fluid pressure are caused by degradation of the vascular glycocalyx. a) Confocal microscopy images of tumoroid MVNs perfused with fluorescent trastuzumab. The arrows indicate focal leaks. The scale bar is 400 µm. b) Permeability to fluorescent dextran, trastuzumab, and cetuximab of tumoroid MVNs compared to control MVNs and c) effective permeability of those MVNs as a function of applied intravascular pressure; *n* = 3. d) Schematic diagram of the computational model of the tumoroid MVNs in the microfluidic device (left), and model results of interstitial fluid pressure, IFP, as a function of changes in vascular hydraulic conductivity, *L*_p_, and matrix permeability, *k*. Typical orders of magnitude for the values in tumors in vivo are indicated. e) Confocal microscopy image of vascular HA in control and SKBR3 MVNs. f) Expression of HA‐associated genes in ECs; *n* = 3. Missing bars in this figure indicate a nondetectable *HAS2* gene expression. g) Quantification of vascular HA concentration in 1 mm biopsies taken 2 mm from the tumoroids in the MVN devices. h) Effective permeability of MVNs subjected to HA‐ase, resulting in increased filtration and hydraulic conductivity; *n* = 3. Significance assessed by one‐way ANOVA after confirming a normal distribution of the data; *p* < 0.05 *, *p* < 0.01 **, *p* < 0.001 ***.

We have previously shown that the MVNs in the vicinity of TC aggregates can partially lose vascular barrier function, resulting in higher permeability across the endothelium.^[^ ^23^ ^]^ We observed the same phenomenon for the breast tumoroid models here, where focal leaks were seen in the MVNs surrounding all three cancer cell line tumoroids (**Figure** **2**a). This loss of junctional integrity resulted in increased MVN permeability in the vicinity of the SKBR3 and MDA‐MB‐468 tumoroids, as measured by the diffusion of dextran (a large model molecule of 70 kDa often used to benchmark vascular permeability^[^ ^30^ ^]^), as well as therapeutic monoclonal antibodies (mABs) trastuzumab (HER2/ERBB2‐targeting)^[^ ^31^ ^]^ and cetuximab (EGFR‐targeting),^[^ ^32^ ^]^ which are used in the treatment of breast cancer (Figure 2b).

The loss of vascular barrier function might intuitively be associated with increased drug delivery to the tumor microenvironment, as higher concentrations of therapeutic molecules would be expected to go through the leaky endothelium and reach the TCs. However, trans‐vascular drug transport is in fact impaired in the tumor microenvironment, and our models capture the additional factors that contribute to this counterintuitive phenomenon. First, the morphology of the MVNs was altered in the vicinity of the tumoroids, as evidenced by a loss of vessel density and specific surface area available for drug transport in the SKBR3 and MDA‐MB‐468 MVNs (Figure S5a, Supporting Information). Second, these morphological alterations also resulted in a loss of MVN perfusion capacity as we observed lower dextran concentrations in the vessels close to the tumoroids compared to vessels at a distance >5 mm in the same MVN devices (Figure S5b, Supporting Information). A third and key determinant of impaired drug delivery was the elevated IFP in the tumoroid microenvironments. This increase in IFP near the tumoroids was assessed by comparing dextran permeabilities between control and tumoroid MVNs under intravascular pressure. When control MVNs are subjected to increasing intravascular pressure, the effective dextran permeability normally increases with applied pressure.^[^ ^33^ ^]^ However, this trend was not observed in the tumoroid MVNs, especially with MDA‐MB‐468 tumoroid MVNs, and the effective permeability at 1500 Pa intravascular pressure was comparable to or lower than in control MVNs (Figure 2c). This finding indirectly shows that the IFP in the vicinity of the tumoroids is also high. As a result, the pressure difference across the endothelium, which drives trans‐vascular fluid flow and additional molecular transport,^[^ ^34^ ^]^ is lower in the tumoroid MVNs compared to control MVNs. This was also evidenced by the lack of interstitial fluid flow in the close vicinity of the tumoroids (Figure S5c, Supporting Information) and the lack of vessel diameter expansion near the tumoroids under applied intravascular pressure (Figure S5d, Supporting Information). Together with the lower intravascular drug concentration and lower vessel surface area, the elevated IFP in the tumoroid microenvironment decreases overall drug transport across tumoroid MVNs despite the increased permeability of unpressurized vessels.

It is well understood that increased ECM density in the desmoplastic stroma contributes to increased IFP through low matrix permeability, *k*, and resistance to interstitial flow.^[^ ^9^, ^30^ ^]^ HA strongly contributes to this phenomenon due to its binding and retention of water molecules.^[^ ^35^ ^]^ Another, less understood contributor to elevated IFP is the leakiness of the vasculature, represented by an increased vascular hydraulic conductivity, *L*_p_. We created a 1D computational model of the tumoroid MVN devices to assess the relevant contribution of *k* and *L*_p_ to IFP in the vicinity of the tumoroids (Figure 2d and Figure S6, Supporting Information). Typical tumor values of *k* and of *L*_p_ are ≥10^−17^ m^2^ and ≥10^−11^ m s^−1^ Pa^−1^, respectively^[^ ^36^, ^37^ ^]^ (Figure 2d). The model results showed that, starting from these known values, an *L*_p_ increase by one order of magnitude can have a greater impact on IFP than a *k* decrease by one order of magnitude (≈160 Pa compared to ≈90 Pa increase in IFP, respectively) (Figure 2d), warranting additional attention to the tumoroid MVNs and the causes of their higher permeability.

Vascular *L*_p_ depends on EC junction integrity and the presence of a functional glycocalyx.^[^ ^34^ ^]^ We did not observe changes in the expression of genes associated with EC junctions between control and tumoroid MVNs (Figure S7a, Supporting Information). However, immunostaining of the marker ZO‐1 revealed wider EC junctions near the tumoroids (Figure S7b, Supporting Information), consistent with previous observations of EC junctions in tumors in vivo.^[^ ^38^ ^]^ Importantly, we observed that vascular HA in the vicinity of the tumoroids was severely degraded (Figure 2e). This is relevant because, as a component of the vascular glycocalyx, HA resists trans‐vascular fluid flow as well as the passage of macromolecules.^[^ ^34^, ^39^ ^]^ The gene expression of *HAS2*, which produces glycocalyx‐associated HA,^[^ ^40^ ^]^ was repressed in ECs near the tumoroids compared to ECs in control MVNs (Figure 2f). Conversely, *HYAL2* expression was increased (Figure S7c, Supporting Information), similar to *CD44* expression (Figure 2f), as previously observed in the tumor microenvironment.^[^ ^41^ ^]^ Vascular glycocalyx degradation was further confirmed by a severe decrease in HA concentration in the periphery of SKBR3 and MDA‐MB‐468 tumoroids (between 0.5 mm and 1.5 mm from the tumoroids) compared to control MVNs (Figure 2g). Degradation of vascular HA after intervention with hyaluronidase (HA‐ase) in SKBR3 tumoroid MVNs did not alter permeability to dextran, while the same intervention increased permeability in control MVNs (Figure S7d, Supporting Information). Importantly, the effective permeability of control MVNs subjected to HA‐ase increased more dramatically with increasing intravascular pressure compared to untreated control MVNs (Figure 2h). Moreover, HA‐ase intervention in control MVNs increased *L*_p_ (here, the gradient of the linear increase) by one order of magnitude, from approximately 10^−12^ to 10^−11^ m s^−1^ Pa^−1^.

These results confirm that vascular HA plays an important role in maintaining a low *L*_p_ in control MVNs, and that loss of vascular HA in tumoroid MVNs is associated with a concurrent increase in *L*_p_, hence in IFP. Despite its partial role in elevated IFP, loss of vascular HA may be used as a marker for cancer‐associated vascular dysfunction. For this reason, we next assessed possible causes for this change in the tumoroid MVNs.

### 2.3 Inhibition of IL8 Restores Endothelial Barrier Function and Enhances Drug Delivery

Vascular dysfunction in the tumoroid MVNs likely results from mechanical and/or biochemical cues specific to the remodeled microenvironment. Contact with a denser, stiffer matrix can disrupt EC junctions.^[^ ^42^ ^]^ Additionally, the lack of fluid flow in poorly perfusable tumoroid MVNs may decrease glycocalyx expression.^[^ ^43^ ^]^ We observed increased *HAS2* expression (Figure 2f) and increased vascular HA concentration (Figure S7e, Supporting Information) in MVNs subjected to physiological vascular flow for 48 hours compared to static MVNs. These changes were consistent with a decreased MVN permeability to dextran (Figure S7d, Supporting Information). However, we have previously observed a functional vascular glycocalyx even under static MVN culture conditions,^[^ ^44^, ^45^ ^]^ as confirmed here by measurable expression levels for *HAS2* in static MVNs (Figure 2f). Moreover, flow‐induced changes in permeability or vascular HA concentration were not observed in the vicinity of the tumoroids (Figure S7d,e, Supporting Information). Thus, impaired vascular flow in the tumor microenvironment may only be partially responsible for the degradation of the vascular glycocalyx. In addition to mechanical cues, pro‐inflammatory cytokines like tumor necrosis factor alpha (TNFα) have previously been shown to induce rapid glycocalyx shedding in ECs.^[^ ^46^ ^]^ We therefore characterized the pro‐inflammatory milieu in the tumoroid MVNs to explore its role in the loss of vascular HA.

**Figure 3:**
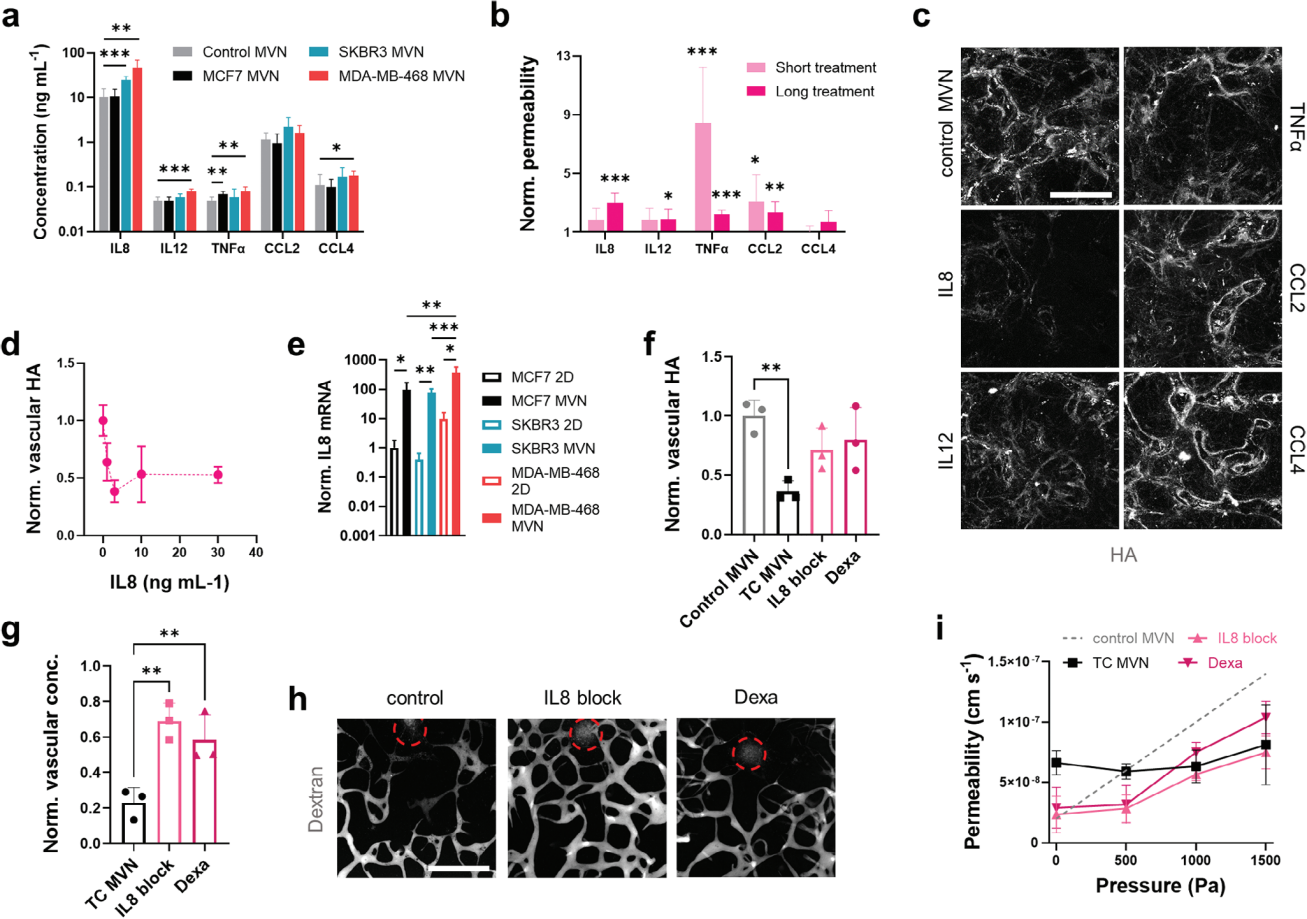
Proinflammatory cytokines in the tumoroid microenvironment contribute to loss of vascular glycocalyx. a) Quantification of pro‐inflammatory cytokines in 1 mm biopsies centered at tumoroids in the MVN devices; *n* = 3. b) MVN permeability after short (15 min) and long (12 h) exposure to proinflammatory cytokines, normalized to untreated MVN permeability; *n* = 3. c) Confocal images of HA in MVNs after long exposure to the cytokines and d) quantification of vascular HA concentration after long exposure to different concentrations of IL8; *n* = 3. e) Gene expression of *IL8* in TCs cultured in 2D or collected from MVN devices; *n* = 3. f) Quantification of HA protein concentration in 1 mm biopsies centered at MDA‐MB‐468 tumoroids after intervention with an IL8 blocking antibody (“IL8 block”), and dexamethasone (“Dexa”). g) Vascular concentration of dextran near the tumoroids normalized to concentration at a distance > 5 mm, as measured by proxy of fluorescence intensity, and h) representative images of perfused MVNs in the vicinity of MDA‐MB‐468 tumoroids (tumoroid cores in circles) subjected to interventions targeting IL8. The scale bar is 500 µm. i) Effective permeability of MDA‐MB‐468 tumoroid MVNs after different interventions as a function of intravascular pressure; *n* = 3. Significance assessed by one‐way ANOVA after confirming a normal distribution of the data; in (e), significance is plotted only between 2D and MVNs for each TC type; *p* < 0.05 *, *p* < 0.01 **, *p* < 0.001 ***.

Cytokine levels in TC conditioned media were first assessed with a broad proinflammatory cytokine array (Figure S8a, Supporting Information). MCF7 TCs appeared to secrete lower concentrations of proinflammatory cytokines compared to SKBR3 and MDA‐MB‐468 TCs. We identified five cytokines with the largest changes in concentration between MCF7 TCs and SKBR3 or MDA‐MB‐468 TCs: IL8, IL12, TNFα, and chemokine ligands 2 and 4 (CCL2/MCP1, CCL4/ MIP‐1β) (Figure S8a, Supporting Information). Subsequent quantification of these cytokines in the lysed tumoroid microenvironments revealed higher concentrations of IL8 in both SKBR3 and MDA‐MB‐468 tumoroid MVNs relative to MCF7 tumoroid MVNs or control MVNs (14 and 35 ng mL^−1^ higher than control MVNs, respectively, **Figure** **3**a). MDA‐MB‐468 tumoroid MVNs also presented higher concentrations of IL12, TNFα, and CCL4 compared to control MVNs (Figure 3a), consistent with the more pro‐inflammatory milieu expected in later stages of breast cancer desmoplasia.^[^ ^47^ ^]^ These increased cytokine concentrations were local to the tumoroids, as analysis of the supernatant collected from the MVN devices revealed much smaller changes relative to control MVNs (Figure S8b, Supporting Information).

We next set out to establish whether these cytokines directly contribute to vascular HA degradation. Recombinant versions of the five cytokines were perfused through control MVNs at a concentration of 5 ng mL^−1^ to assess changes in permeability after short (<15 min) and long (12 h) exposure times. TNFα and CCL2 increased MVN permeability to dextran after a short exposure, while significant changes in permeability were produced by a long exposure with IL8, IL12, TNFα, and CCL2, with IL8 showing the largest increase in permeability (from 1.9 × 10^−8^ to 5.7 × 10^−8^ cm s^−1^, Figure 3b). End‐point analysis of vascular HA concentration by immunofluorescence showed significant HA degradation by CCL2 after a short exposure (Figure S8c, Supporting Information) and by IL8 after a long exposure (Figure 3c). Exposure of control MVNs to different concentrations of IL8 confirmed the loss of vascular HA at a concentration as low as 1 ng mL^−1^ (Figure 3d). Interestingly, *IL8* gene expression increased in all tumoroid MVNs types compared to 2D culture (Figure 3e). IL8 has angiogenic effects,^[^ ^48^ ^]^ and its expression in TCs may be enhanced by paracrine signaling from ECs in the tumor microenvironment. Overall, these results point to IL8 as a target cytokine to prevent vascular HA degradation and subsequent loss of vascular barrier function.

We tested this hypothesis by subjecting MDA‐MB‐468 tumoroid MVNs to interventions targeting IL8: an IL8‐blocking monoclonal antibody (mAB), and the broad anti‐inflammatory small molecule dexamethasone. All interventions were administered in the MVN devices over 4 d. We first assessed changes in vascular HA concentration and found that both the IL8‐blocking mAB and dexamethasone aided in the recovery of vascular HA expression to levels similar to control MVNs (0.7‐ and 0.8‐fold relative to controls, respectively, Figure 3f). The two molecules also improved MVN perfusion near the tumoroids (intravascular dextran concentration measured as 0.7‐ and 0.6‐fold relative to distant controls for IL8‐blocking mAB and dexamethasone, respectively, Figure 3g,h). The interventions decreased tumoroid MVN permeability to dextran to values comparable to control MVNs (2.3 × 10^−8^ and 2.9 × 10^−8^ cm s^−1^ for IL8‐blocking mAB and dexamethasone, respectively, Figure 3i) and increased effective permeability with applied intravascular pressure (Figure 3i), indicating a decrease in vascular *L*_p_, and hence a decrease in IFP. Nevertheless, the lower effective permeabilities measured in the tumoroid MVNs subjected to interventions compared to healthy controls confirm the persistence of a low matrix *k* in the tumoroid microenvironments.

These results identify IL8 as an attractive target to aid in the recovery of the vascular glycocalyx as a way of increasing drug penetration and delivery in breast tumors. Importantly, the results also suggest that targeting pathophysiological mechanisms affecting the vascular ECM can provide a different strategy to normalize the desmoplastic tumor microenvironment other than targeting the stromal ECM. To compare the effects of these two therapeutic strategies, we next assessed potential interventions to degrade stromal HA.

### 2.4 Degradation of Stromal Hyaluronic Acid Enhances Drug Delivery and Tumor Cell Death

**Figure 4:**
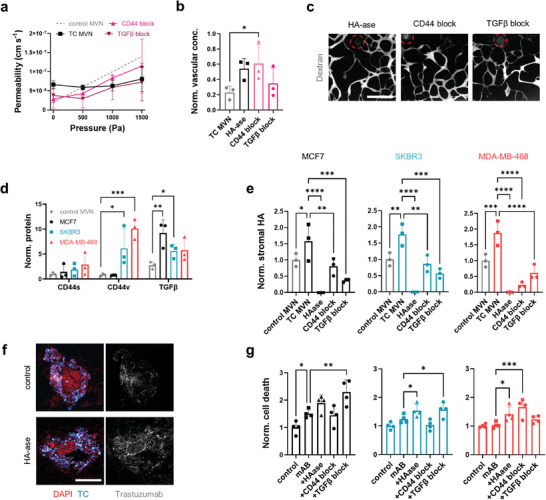
Degradation of stromal HA increases drug penetration and TC death in the tumoroids. a) Effective permeability of MDA‐MB‐468 tumoroid MVNs after different interventions targeting stromal HA as function of intravascular pressure; *n* = 3. b) Vascular concentration of dextran near the tumoroids normalized to the concentration at a distance > 5 mm after intervention with HA‐ase, a CD44 blocking antibody (“CD44 block”), and a TGFβ blocking antibody (“TGFβ block”), as measured by proxy of fluorescence intensity, and c) representative confocal images of tumoroid (circles) MVNs perfused with dextran after different interventions. d) Quantification of CD44 isoforms and TGFβ in 1 mm biopsies centered at the tumoroids in the MVN devices, and e) quantification of stromal HA concentration in the tumoroids after stromal HA‐targeting interventions. f) Confocal microscopy images of SKBR3 tumoroid cryosections showing increased trastuzumab penetration after HA degradation. g) Cell death in the tumoroids as a function of combined treatment with trastuzumab + cetuximab (“mAB”) with different strategies targeting stromal HA, as measured by proxy of fluorescent SYTOX intensity. Significance assessed by one‐way ANOVA after confirming a normal distribution of the data; *p* < 0.05 *, *p* < 0.01 **, *p* < 0.001 ***, *p* < 0.0001 ****.

We subjected MDA‐MB‐468 tumoroids MVNs for 4 d to HA‐ase, a CD44‐blocking mAB, and a TGFβ‐blocking mAB. While HA‐ase and the CD44‐blocking mAB target stromal HA directly by degrading it or preventing TCs and FBs from binding to it, respectively, the TGFβ‐blocking mAB indirectly targets HA by depriving FBs in the tumoroids of TGFβ, a key stimulant of stromal HA production.^[^ ^49^ ^]^ Similar to the interventions targeting IL8, blocking CD44 and TGFβ in the tumoroid stroma decreased MVN permeability (2.9 × 10^−8^ cm s^−1^ for CD44 blocking, 3.8 × 10^−8^ cm s^−1^ for TGFβ blocking, **Figure** **4**a) and IFP, as seen by an increase in effective MVN permeability with applied intravascular pressure (Figure 4a). This effect was particularly pronounced for the CD44‐blocking intervention, as effective permeability values were only slightly lower than permeability values of control MVNs. HA‐ase, instead, did not lower MVN permeability (5.9 × 10^−8^ cm s^−1^), but rather increased effective permeability under intravascular pressure to levels higher than control (Figure S8e, Supporting Information), likely the result of further degradation of vascular HA in addition to stromal HA. On average, all three interventions improved MVN perfusion near the tumoroids, with CD44 blocking showing the only statistically significant increase in vascular dextran concentration relative to untreated tumoroid MVNs (approximately 0.6‐fold relative to concentration levels at distant sites, Figure 4b,c). These results indicate that targeting the tumoroid stromal ECM can improve vascular function through a decrease in vessel constriction by the dense ECM.^[^ ^50^, ^51^ ^]^ In particular, the decrease in tumoroid IFP, hence the increase in transvascular flow, was better achieved by targeting stromal ECM rather than targeting vascular ECM. This is likely due to the additional increase in matrix permeability *k* when stromal HA is degraded.

Because the three tumoroid types showed differential expression and localization of HA, we next assessed how the interventions affect stromal HA concentration in the different tumoroid types by measuring protein expression of CD44v (the CD44 isoform associated with TC invasion^[^ ^45^, ^52^ ^]^), the more benign isoform CD44s, and TGFβ. We found increased protein expression of CD44v in the SKBR3 and, more pronouncedly, in the MDA‐MB‐468 tumoroids relative to controls MVNs (Figure 4d). Conversely, the level of CD44s was relatively low and constant between groups (Figure 4d). TGFβ expression was high in all three tumoroid types, though the highest in MCF7 tumoroids (Figure 4d). These different target concentrations in the tumoroids likely affected the response to the different interventions. All three interventions successfully and severely decreased stromal HA concentration in the tumoroids, although TGFβ blocking was more efficient than CD44 blocking in the MCF7 and SKBR3 tumoroids, while the opposite was true in the MDA‐MB‐468 tumoroids (Figure 4e). The greatest effect was achieved by HA‐ase, which fully degraded HA in all three tumoroid types.

We further hypothesized that degradation of stromal HA can lead to increased drug delivery to TCs. To test this, the tumoroid MVNs were treated for 4 d with trastuzumab and cetuximab, a model drug combination with expected cytotoxic effects.^[^ ^31^, ^32^ ^]^ The drugs were administered at a concentration of 20 µg mL^−1^ to match the expected levels in circulation.^[^ ^33^ ^]^ Data obtained with tumoroids in well‐plates showed significant TC death in all tumoroid types when trastuzumab and cetuximab were administered in combination at 20 µg mL^−1^, as measured by fluorescence intensity of a cell death marker (Figure S9c, Supporting Information). We used the same method to evaluate cell death as a result of co‐perfusion of trastuzumab and cetuximab with the interventions targeting stromal HA in the MVN devices. Sections of the fixed tumoroids after drug treatment revealed a qualitative increase in drug penetration after HA‐ase intervention compared to controls (Figure 4f), supporting our hypothesis that stromal HA degradation increases interstitial drug transport. When perfusing trastuzumab and cetuximab without interventions targeting HA, significant cell death was only observed in the MCF7 tumoroids relative to untreated controls, despite the relatively high drug concentration perfused (Figure S9a, Supporting Information and Figure 4g). This confirmed the negative effect of impaired vascular perfusion and increased ECM density on drug delivery in the other tumoroid types. Intervention with HA‐ase in addition to drug treatment increased TC death in SKBR3 and MDA‐MB‐468 tumoroids, while intervention with the TGFβ‐blocking mAB increased TC death in MCF7 and SKBR3 tumoroids. Moreover, intervention with the CD44‐blocking mAB increased TC death in MDA‐MB‐468 tumoroids. Measurements of tumoroid size changes were less conclusive, as a significant decrease in size over the 4 d could only be observed with the HA‐ase intervention (Figure S9b, Supporting Information), likely due to its particularly efficient degradation of stromal HA. Overall, these results confirm our hypothesis that stromal HA degradation in the tumoroid microenvironments is associated with increased drug efficacy due to improved penetration. The results also showcase the capability of the models to assess the relative impact of different therapeutic strategies in different tumoroid models. We next leveraged this to test therapeutic strategies in vascularized tumoroids assembled from patient‐derived TCs.

### 2.5 Personalized Tumoroid Models Capturing Microenvironmental Heterogeneity Can Screen Desmoplasia Normalization Strategies

**Figure 5:**
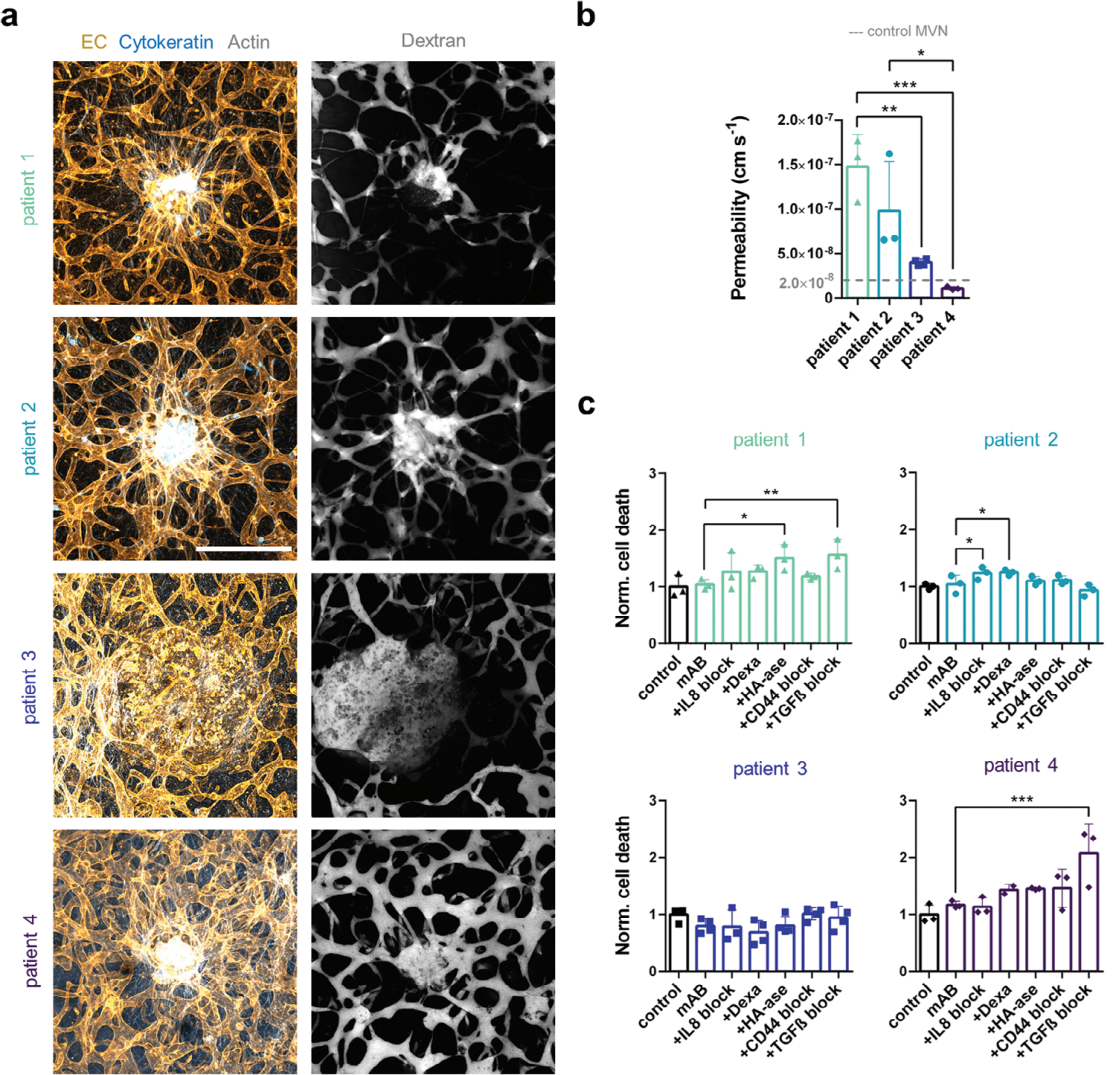
Vascularized tumoroids from patient‐derived breast TCs respond differently to interventions normalizing vascular and stromal HA. a) Projected confocal microscopy images of patient‐derived tumoroids show distinct levels of vascularization and TC invasion. b) Permeability of patient‐derived tumoroid MVNs; *n* = 3–4. c) Cell death in the patient‐derived tumoroids as a function of combined treatment with trastuzumab + cetuximab (“mAB”) with different strategies targeting vascular and stromal HA, as measured by proxy of fluorescent SYTOX intensity; *n* = 3–4. Significance assessed by one‐way ANOVA after confirming a normal distribution of the data; *p* < 0.05 *, *p* < 0.01 **, *p* < 0.001 ***.

We formed tumoroids with four breast cancer patient‐derived cell types (luminal: Patient 1, basal: Patients 2 and 4; metastatic: Patient 3) with differential expression of therapeutic target receptors (Figure S10, Supporting Information). TCs from Patient 1 were HER2+/EGFR‐ (similarly to SKBR3 cells). TCs from Patient 2 and Patient 3 were HER2+/EGFR+, while TCs from Patient 4 were HER2‐/EGFR‐ (similarly to MCF7 cells). Patient‐derived tumoroids were cultured within MVN devices (**Figure** **5**a) and became more vascularized by the MVNs compared to cancer cell line tumoroids (Figures 1a and 2a). However, the level of vascularization seemed to depend on the TC type. Luminal TC tumoroids (from Patient 1) were only partially vascularized, while basal TC tumoroids (from Patients 2 and 4) were fully vascularized, as shown by fluorescent dextran perfusion (Figure 5a). TCs from Patient 2 were particularly invasive and actively invaded the tumoroid MVNs (Figure 5a). Remarkably, metastatic TC tumoroids (from Patient 3) aggressively degraded the surrounding tissue, creating a cyst‐like hollow structure where dextran pooled (Figure 5a). Patient‐derived tumoroid MVNs further displayed heterogeneity in terms of vascular barrier function, as measured by a wide range of permeability to dextran (from 1.1 × 10^−8^ cm s^−1^ in tumoroid MVNs from Patient 4 to 1.5 × 10^−7^ cm s^−1^ in tumoroid MVNs from Patient 1) (Figure 5b). Interestingly, the permeability of tumoroid MVNs from Patient 4 was similar to the permeability of MCF7 MVNs, and the permeability of tumoroid MVNs from Patient 1 was similar to the permeability of SKBR3 MVNs (Figure 2b). These results show that our models can be used with cells derived from different cancer types and patients to capture breast cancer heterogeneity.

Finally, as a proof‐of‐concept, we used patient‐derived tumoroid MVN models to test the cytotoxic effect of trastuzumab and cetuximab in combination with the different interventions targeting vascular and stromal HA, as previously done with the cancer cell line tumoroids. We found that tumoroids derived from Patient 1 and Patient 4 were more vulnerable to drug treatment in combination with different interventions compared to tumoroids from Patient 2 and Patient 3, as measured by higher TC death (Figure 5c). In tumoroids derived from Patient 1, cell death increased significantly when cytotoxic drugs were perfused in combination with HA‐ase or the TGFβ‐blocking mAB (Figure 5c). Interestingly, a similar response was observed in SKBR3 tumoroids (Figure 4g), which also expressed HER2/ERBB2 but not EGFR (Figure S1a, Supporting Information). Intervention with the TGFβ‐blocking mAB also considerably increased cell death in tumoroids derived from Patient 4 (1.9‐fold relative to tumoroids only treated with trastuzumab and cetuximab) (Figure 5c). This was in line with the response of MCF7 tumoroids (Figure 4g), which were also double‐negative for HER2/ERBB2 and EGFR (Figure S1a, Supporting Information). Despite the lower efficacy of trastuzumab and cetuximab in tumoroids derived from Patient 2 tumoroids, we observed increased cell death when the tumoroids were additionally subjected to the IL8‐blocking mAB or dexamethasone (Figure 5c). Interestingly, tumoroids derived from metastatic TCs from Patient 3 were non‐responsive to all the treatment combinations (Figure 5c). Taken together, these results show that patient‐derived vascularized tumoroid models can be used to test how different strategies that normalize the tumoroid microenvironment improve drug delivery.

## 3 Discussion

In this study, we first aimed to recapitulate in vitro the biomechanical microenvironment of breast cancer desmoplasia, which leads to impaired drug delivery to the tumor. Tumoroids formed with breast cancer cell lines (MCF7, SKBR3, or MDA‐MB‐468) and FBs were cultured in MVNs in microfluidic devices. Our results show that tumoroid MVNs containing more invasive TC types (SKBR3 and MDA‐MB‐468 cells) present higher HA deposition and enhanced transformation of FBs into CAFs (αSMA‐positive fibroblasts) relative to MCF7 tumoroid MVNs or control MVNs (Figure 1). Particularly in MDA‐MB‐468 MVNs, these findings are associated with a high expression of *HAS2* in TCs and *CD44* in both TCs and FBs (Figure 1). These results demonstrate the ability of our vascularized tumoroid models to capture the variations in desmoplastic ECM deposition depending on the tumoroid type and agree with previous pre‐clinical and clinical data. For example, it was previously shown that a bone‐metastasizing breast cancer cell line cultured in 2D produced more HA via enhanced *HAS2* expression compared to its parental cell line.^[^ ^53^ ^]^ We further show increased *HAS2* expression in MDA‐MB‐468 MVNs relative to MDA‐MB‐468 cultured in 2D, pointing towards a better recapitulation of the 3D breast cancer microenvironment. In fact, clinical data show that HA expression is increased in malignant breast tumors, especially in the peritumoral stroma, and is a prognostic factor of patient overall survival.^[^ ^54^ ^]^

Our models also recapitulate impaired vascular barrier function and perfusion, as evidenced by elevated vascular permeability and IFP in the vicinity of SKBR3 and MDA‐MB‐468 tumoroids MVNs compared to control MVNs (Figure 2). Remarkably, the nearly threefold increase in dextran permeability in our models (2.8 × 10^−8^ cm s^−1^ in control MVNs versus 8.1 × 10^−8^ cm s^−1^ in SKBR3 MVNs) is comparable with measurements in tumors in vivo (factor of ≈6).^[^ ^30^ ^]^ Moreover, the observation that high IFP impairs vascular perfusion in our models reflects the phenomenon seen in desmoplastic breast cancer, where increased IFP hinders drug penetration into the tumor and negatively correlates with patient survival.^[^ ^9^, ^30^, ^55^ ^]^ Taking into account both vascular permeability and IFP, strategies that aim to normalize the tumor vasculature may impact drug delivery in two opposite but simultaneous ways: on one hand, enhancing it by lowering tumor IFP, and on the other hand, potentially hindering it by decreasing vascular permeability and drug transport across the endothelium. Our vascularized tumoroid models enable a better understanding of these phenomena and can be used to test the effect of potential normalization strategies to improve drug delivery to the tumor. When investigating the cause of impaired vascular barrier function and perfusion, we found increased vascular HA degradation in SKBR3 and MDA‐MB‐468 tumoroids MVNs compared to control MVNs (Figure 2). These results align with a clinical study showing an impairment of endothelial glycocalyx integrity in cancer patients that was proportional to cancer stage^[^ ^56^ ^]^ Endothelial glycocalyx degradation was also shown to play a role in cancer metastasis (reviewed in^[^ ^57^ ^]^). For example, we have previously shown in our engineered microvascular models that vascular HA degradation not only impairs vascular barrier function but also favors TC arrest in the vasculature and extravasation.^[^ ^45^ ^]^

It is tempting to speculate that interventions that restore vascular HA while degrading stromal HA may be particularly beneficial for normalizing the breast cancer microenvironment and preventing disease progression. To test the molecular mechanisms responsible for low vascular HA levels, impaired vascular perfusion, and permeability, we analyzed the secretion of pro‐inflammatory cytokines in tumoroid MVNs. We identified IL8 as a potential target to normalize vascular HA levels, as its secretion was increased in SKBR3 and MDA‐MB‐468 tumoroids MVNs compared to control MVNs (Figure 3). Moreover, treating control MVNs with recombinant IL8 increased their permeability and reduced vascular HA (Figure 3), thus mimicking the effects observed in tumoroid MVNs relative to control MVNs. Our results echo the finding that IL8 concentration is significantly increased in the serum of patients with advanced breast cancer, correlating negatively with patient survival.^[^ ^58^ ^]^ Indeed, IL8 can increase the invasiveness and metastatic potential of breast TCs (reviewed in^[^ ^59^ ^]^). We found that interventions with an IL8‐blocking mAB or with the broad anti‐inflammatory drug dexamethasone partially restored normal levels of vascular HA, perfusion, and permeability (Figure 3). Interventions targeting IL8 and the pro‐inflammatory tumor milieu may find a new use in normalizing breast cancer desmoplasia, as exemplified by dexamethasone, which was recently shown to increase stromal and vascular normalization in murine breast cancer models.^[^ ^60^ ^]^

While vascular HA degradation in the tumor microenvironment impairs vascular barrier function and perfusion, stromal HA degradation can improve interstitial transport of therapeutic molecules to TCs. In addition to testing strategies that target the vasculature, we thus tested interventions that normalize the tumor stroma and improve drug delivery in the cancer cell line tumoroid models. We found that interventions targeting stromal HA restored vascular permeability, reduced IFP, and improved vascular perfusion (Figure 4). These findings are clinically relevant, as the normalization of desmoplastic tumors is currently being targeted therapeutically by modulating the cell contractility of TCs and CAFs or their aberrant ECM deposition,^[^ ^10^ ^]^ thus preventing blood vessel constriction and improving drug penetration. We directly assessed the effect of HA‐targeting interventions on TC death as we perfused the tumoroid MVNs with trastuzumab and cetuximab and showed that drug delivery can be restored (Figure 4). Among these interventions, HA‐ase, in the relatively high concentration used here, resulted in the degradation of both vascular and stromal HA in the tumoroid microenvironments, likely producing exaggerated fluid filtration that may result in edema in vivo. However, we show that interventions targeting TGFβ improve drug delivery in early‐stage breast cancer (MCF7 and SKBR3) tumoroid MVNs. Targeting TGFβ appears particularly attractive, as TGFβ stimulates desmoplastic collagen and HA deposition.^[^ ^61^ ^]^ In fact, in murine breast carcinoma models, TGFβ blocking was found to decrease collagen I content in the tumor stroma and increase vascular perfusion, thus improving drug delivery.^[^ ^62^ ^]^ We additionally show that preventing HA binding by blocking CD44 can also normalize the tumor microenvironment and restore drug delivery, as observed in our models of advanced, aggressive breast cancer (MDA‐MB‐468 MVNs), which also expressed the highest level of CD44 (Figure 4). This observation is consistent with the identification of HA and CD44 as markers of metastatic breast cancer,^[^ ^52^, ^63^ ^]^ and supports the notion that inhibiting their activity can prevent TC dissemination.^[^ ^45^ ^]^

Importantly, the MVN models enable the culture of patient‐derived tumoroids in functional microvascular beds that can be perfused with relevant therapeutic molecules under physiological vascular flow. We found that our models recapitulated heterogeneity between patients in terms of variability in tumoroid vascularization, vascular permeability, as well as response to different interventions targeting the desmoplastic microenvironment (Figure 5). Interestingly, vascularization seems to depend on tumor type (luminal, basal, or metastatic) while vascular permeability and response to normalizing interventions seem to be associated with the expression of HER2/ERBB2 and EGFR. For example, our results show increased vascularization of tumoroids derived from basal TCs (from Patients 2 and 4) (Figure 5). Our observations agree with the results of previous studies showing that basal phenotypes are associated with higher microvascular density and microvessel proliferation relative to non‐basal or luminal phenotypes in breast cancer.^[^ ^64^, ^65^, ^66^, ^67^ ^]^ In terms of responses to normalizing interventions, targeting TGFβ in vascularized tumoroid models appears to be a successful strategy to improve drug delivery, particularly with TCs lacking EGFR (MCF7, SKBR3, Patient 1, and Patient 4). We speculate that this might be due to less ECM accumulation in these models before intervention relative to models containing EGFR+ TCs. While the IL8‐blocking mAB and dexamethasone interventions were effective in improving drug delivery in tumoroids derived from Patient 2, we did not observe an effect of the CD44 blocking‐intervention on drug‐induced cytotoxicity in our patient‐derived samples (Figure 5). We speculate that patient‐derived TCs might compensate for the blockade of CD44 by over‐expressing other HA‐binding receptors such as CD168, which is associated with breast cancer invasion.^[^ ^68^ ^]^

It is important to acknowledge that the clinical relevance of our observations in patient‐derived models is preliminary, as tumoroids were derived from only four patients. Moreover, only a limited set of experiments could be performed with TCs derived from each patient given the difficulty of expanding them in high numbers. Our observations need to be confirmed in future studies with a larger sample size and in vitro data need to be directly compared to clinical data. Moreover, future studies could focus on further improving the pathophysiological relevance of the models. For example, primary human umbilical vein ECs (HUVECs) and lung FBs were chosen for their vasculogenic potential and robust formation of perfusable MVNs even when exposed to pro‐inflammatory factors.^[^ ^23^, ^45^ ^]^ Future versions of the models may include immortalized MVN cells sources with improved reproducibility,^[^ ^69^ ^]^ induced pluripotent stem cell‐derived ECs, or primary mammary ECs and FBs cultured under constant vascular flow for improved physiological tissue fidelity and model longevity.^[^ ^70^ ^]^ Additionally, to capture intratumor heterogeneity, tumoroids derived via sampling different parts of an excised tumor may also be tested in isolation. Patient‐derived, organotypic tumoroids that include native immune cell populations and ECM may also be incorporated in the models to assess the efficacy of immunotherapies.^[^ ^13^ ^]^

## 4 Conclusion

The heterogeneity within breast cancer desmoplasia underscores the significance of utilizing patient‐specific in vitro models to precisely identify treatment strategies that yield optimal therapeutic responses. The vascularized breast tumoroids presented here capture key elements of microenvironmental heterogeneity that impair drug delivery to TCs. These aspects depend on the invasiveness of the TCs used to form the tumoroids. Models containing more invasive TC types (SKBR3 and MDA‐MB‐468 cells) showed increased levels of stromal HA deposition, vascular permeability, IFP, and degradation of vascular HA relative to models containing MCF7 tumoroids or models without tumoroids. IL8 secretion was identified as a factor responsible for vascular dysfunction and loss of vascular HA. Interventions targeting IL8 or stromal HA restored normal levels of vascular permeability, perfusion, IFP, and improved drug delivery as measured by increased TC death in response to perfusion with the cytotoxic drugs trastuzumab and cetuximab. Similar responses were observed in our vascularized models when used with patient‐derived tumoroids to assess the efficacy of various therapeutic approaches in a patient‐specific manner. These models hold promise as personalized translational assays, offering valuable insights into clinical therapeutic strategies and facilitating the identification of those with a heightened likelihood of treatment success.

## 5 Experimental Section

### Vascularized Tumoroid Formation

Cancer cell lines MCF7, SKBR3, and MDA‐MB‐468 were obtained from ATCC and cultured in Dulbecco's modified Eagle's medium (#10566016, ThermoFisher) supplemented with 10% fetal bovine serum (#12662029, ThermoFisher). All cancer cell lines were made to express red fluorescent protein (RFP) as previously described.^[^ ^45^ ^]^ Patient‐derived TCs were obtained from ZenBio (#MBE‐F‐TM and #MLE‐F‐TM) and from the Dana‐Farber Cancer Institute Center for Patient Derived Models (#CPDM_1227X) and cultured in mammary epithelial cell growth medium (#C‐21010, Promocell). These TCs were preselected for their ability for expansion and tumoroid formation. The successful rate of tumoroid formation was thus 100%. Human umbilical vein ECs (HUVECs, wild type or GFP‐expressing) from pooled donors were obtained from Angio‐Proteomie (#cAP‐0001 and #cAP‐0001GFP) and cultured in vasculife endothelial medium (#LL‐0003, Lifeline) up to passage 5. Normal human lung FBs were obtained from Lonza (#CC‐2512) and cultured in Fibrolife Fibroblast Medium S2 (#LL‐0011, Lifeline) up to passage 5. Tumoroids were self‐assembled by coculture of 4000 TCs and 5000 FBs in non‐adherent 96‐well plates (PrimeSurface 96 M, Sbio) over 4 d without changing culture medium. MVNs incorporating tumoroids were formed as previously described,^[^ ^23^ ^]^ in a three‐channel microfluidic device (central gel channel: 3 mm x 0.5 mm x 1 cm)^[^ ^33^, ^44^, ^71^ ^]^ made from polydimethylsiloxane (PDMS, Ellsworth Adhesives) bound to no. 1 glass coverslips (#48404‐097, VWR). ECs (6 million mL^−1^), FBs (2 million mL^−1^), and tumoroids were co‐injected with fibrin gel solution within the central channel of the microfluidic device and cultured for 7 d with daily vasculife medium changes in the side channels. A monolayer of ECs was seeded on the gel surfaces in the side channels on day 4 of culture, as previously described.^[^ ^44^ ^]^

### MVN Permeability and Fluid Flows

Permeability of fluorescent molecules (fluorescein isothiocyanate (FITC, #F4274, Sigma Aldrich), dextran (FITC‐conjugated, 70 kDa, 0.1 mg mL^−1^, #FD70, Sigma Aldrich), trastuzumab (Alexa Fluor 488‐conjugated, #FAB9589G, 0.1 mg mL^−1^, R&D Systems), and cetuximab (Alex Fluor 647‐conjugated, #FAB9577R, 0.1 mg mL^−1^, R&D Systems)) in the MVNs was measured by confocal microscopy and images were analyzed with the software ImageJ, as previously described.^[^ ^44^ ^]^ The permeability analysis also yields the morphological parameters measured:^[^ ^44^ ^]^ vascular volume fraction, *V%*, specific surface area, *SSA*, and average vessel diameter, *d*. The MVNs were conditioned with vascular flow for 48 h using a custom pump that applies a constant pressure difference across the MVNs of approximately 50 Pa.^[^ ^72^ ^]^ Pressurization of the MVNs through the microfluidic device side channels was done as shown previously,^[^ ^33^ ^]^ using a FlowEZ pressure regulator (Fluigent), and the effective MVN permeability for intravascular pressures up to 1500 Pa was measured as described above. The increase in effective permeability of FITC was used to calculate the hydraulic conductivity of the MVNs, *L*_p_, using Equation 1:^[^ ^73^ ^]^

(1) $$P_{\mathrm{eff}}=P+L_{p}\;\Delta p$$

where *P* is the MVN permeability at intravascular pressure Δ*p* = 0 Pa and *P*_eff_ is the effective permeability. No endothelial reflection of the small solute and no difference in osmotic pressure across the endothelium were assumed, as described before for this system.^[^ ^33^ ^]^ The interstitial flow resulting from MVN pressurization in the vicinity of the tumoroids was measured by fluorescence tracking of a 30 µm bleached spot, as previously described.^[^ ^33^ ^]^

### Interstitial Fluid Pressure Modeling

Computational modeling of IFP in the MVN devices was performed using the software COMSOL Multiphysics v5.4. The gel channel of the microfluidic device was described by a 1D domain divided into four subsequent zones: the tumor spheroid (A: 0–0.5 mm), the gel areas close to the spheroid (B: 0.5–3 mm) and far from it (C: 3–5 mm), and the exit portion of the chip (D: 5–7 mm). Only half of the chip was simulated due to symmetry, identifying the spheroid center as origin of the domain (*x* = 0, Figure 2d). Fluid flow in the gel was accounted for by the Darcy equation, and the Starling equation was used to include fluid filtration from both the MVNs and the lateral monolayers, neglecting osmotic differences.^[^ ^44^ ^]^ The resulting Equations 2 and 3 were:

(2) $$\frac{dp}{dx}=-\frac{\mu }{k}\;u$$

(3) $$\frac{du}{dx}=\left(\frac{A_{S}}{V}L_{p}+\frac{2}{W}L_{p,M}\right)\;\left(p_{v}-p\left(x\right)\right)$$

where *p* is the fluid pressure, *u* is the fluid velocity, *µ* is the fluid viscosity, *k* is the matrix permeability, *A*_S_/*V* is the ratio of the MVN lateral surface area and the matrix volume, *L*_p,M_ is the hydraulic conductivity of the side monolayer, assumed one order of magnitude higher than that of the MVNs based on the previous assessments,^[^ ^33^ ^]^ and *W* is the width of the gel channel. Boundary conditions complemented the model imposing the symmetry condition (*u*(0) = 0), and the atmospheric pressure as outlet (*p*(7 mm) = 0). Parameter values are reported in Table S1 (Supporting Information). Geometric differences at the exit of the microfluidic device were included in the model as a change of Darcy equation parameters. The relative effect of *L*_p_ and *k* in the *B* area of the device was investigated by changing their values across different orders of magnitude and analyzing resulting fluid pressure and velocity.

### Gene and Protein Expression

Gene expression was assessed by quantitative polymerase chain reaction (qPCR). Tumoroids from 4 microfluidic devices, 4 tumoroids per device, were collected by separating PDMS and glass using a scalpel to access the gel, as previously described.^[^ ^74^ ^]^ Gel biopsy punches (1 mm diameter, #15110‐10, Ted Pella) centered at each tumoroid were collected and the matrix between cells was dissociated with Liberase (5 mg mL^−1^, #5401135001, Sigma Aldrich) for 30 min. ECs (GFP), TCs (RFP), and FBs (non‐labeled) were then resuspended in a flow cytometry buffer (2% fetal bovine serum, 2 × 10^−3^ m EDTA in PBS) and sorted using the BD FACSAria III Cell Sorter. Dead cells were eliminated by DAPI staining. Sorted cells were lysed in Trizol (#15596026, Fisher Scientific), RNA was extracted and reverse‐transcribed into cDNA. Custom PCR plates (TaqMan 96 well‐plate, ThermoFisher) were used to assess mRNA expression on a QuantStudio 12K Flex Real‐Time PCR System (ThermoFisher) for the following genes: *GAPDH*, *EGFR*, *ERBB2*, *PGR*, *ESR1*, *ABCB1*, *ABCG2*, *CXCL8*, *IL12A*, *CCL2*, *CCL4*, *TNF*, *VEGFA*, *FGF1*, *CDH1*, *HAS1*, *HAS2*, *HAS3*, *HYAL1*, *HYAL2*, *CD44*, *TJP1*, *CLDN5*, *OCLN*, *FCGRT*, *CAV1*, *CLTC*. Gene expression was normalized to *18S* and *GAPDH* as endogenous controls. Missing data values represent non‐detectable signals. Protein expression was assessed in four ways: i) immunofluorescence staining of EGFR (cetuximab, Alex Fluor 647‐conjugated, #FAB9577R, 0.1 mg mL^−1^, R&D Systems), HER2 (trastuzumab, Alexa Fluor 488‐conjugated, #FAB9589G, 0.1 mg mL^−1^, R&D Systems), cytokeratin (#M3515, Agilent), HA (HA binding protein, #385911, Sigma Aldrich), αSMA (#19245S, Cell Signaling Technology), HIF‐1α (#ab8366, Abcam), Caspase‐3 (#9661S, Cell Signaling Technology), COL‐1 (#AF6220‐SP, R&D Systems), ZO‐1 (#33‐9100, ThermoFisher); ii) ProteinSimple automated Western analysis, as described previously,^[^ ^45^ ^]^ of EGFR (#MAB9577, R&D Systems), HER2 (#MAB9589, R&D Systems), CD44 (#GTX102111, GeneTex, isoform assessed at peaks CD44s: 90 kDa, and CD44v: 160 kDa), TGFβ (#MAB1835, R&D Systems); iii) Enzyme‐linked immunosorbent assay (ELISA) of HA (#DHYAL0, R&D Systems), normalized to total protein content, and pro‐inflammatory cytokines (array, #ab134003, Abcam); iv) custom Meso Scale Discovery (MSD) assay of proinflammatory cytokines IL8, IL12, CCL2, CCL4, TNFα. Protein quantification was performed on single 1 mm gel biopsy punches lysed in buffer (#9803S, Cell Signaling Technologies) containing benzonase nuclease (#E8263, Sigma Aldrich) and protease inhibitor cocktail (#11836170001, Sigma Aldrich). Immunostaining was performed on either intact gels fixed with 4% paraformaldehyde (#50‐259‐96, Fisher Scientific) within microfluidic devices, or on gels extracted from devices and embedded in Optimal Cutting Temperature (OCT) compound before cryosectioning on a Leica 1850 cryotome.

### Interventions Normalizing the Tumor Microenvironment and Drug Treatments

Interventions were administered in the microfluidic devices through perfusion across the MVNs. HA‐ase (150 units mL^−1^, #H3631, Sigma Aldrich) was perfused for 30 min under a transient pressure difference across the MVNs before measurement of *L*_p_. Recombinant human IL8 (#208‐IL‐010, R&D Systems), IL12 (#219‐IL‐005, R&D Systems), TNFα (#210‐TA‐005, R&D Systems), CCL2 (#279‐MC‐010, R&D Systems), CCL4 (#271‐BME‐010, R&D Systems) were perfused at a concentration of 5 ng mL^−1^ together with dextran for short‐exposure permeability testing (<15 min) and for 12 h for long‐exposure testing. MVN and tumoroid interventions were administered over 4 d through daily media perfusion with an IL8 blocking antibody (20 µg mL^−1^, #MAB208, R&D Systems), dexamethasone (5 × 10^−6^ m, #D4902, Sigma Aldrich), HA‐ase (150 units mL^−1^, #H3631, Sigma Aldrich), a CD44 blocking antibody (10 µg mL^−1^, 08‐9407‐2, American Research Products), and a TGFβ blocking antibody (20 µg mL^−1^, #MAB1835, R&D Systems). Trastuzumab (#MAB9589, R&D Systems) and cetuximab (#MAB9577, R&D Systems) were co‐perfused at a concentration of 20 µg mL^−1^, and changes in cell death in the tumoroids were assessed through imaging of SYTOX Green (#S34860, ThermoFisher) on a Nikon Eclipse Ti microscope with a 4× objective. The SYTOX signal intensity was measured using ImageJ in the area colocalized with the RFP TC signal. Changes in tumoroid size as a result of the different interventions in addition to drug treatment were measured in terms of changes in projected area, also through the RFP TC signal. For the patient‐derived, nonfluorescent TCs, the SYTOX signal intensity was measured in a circular area of 1 mm diameter centered on the tumoroids.

### Statistical Analysis

Data were checked for normality and presented as mean ± SD. When possible without compromising clarity, single biological repeat data points are provided in the graphs. Data were typically collected from 3 devices, with the exact number of technical and biological repeats being reported in each figure caption. Statistical analysis of the data was performed with the software GraphPad Prism (version 9) by typically using a one‐way ANOVA after confirming normal distribution of the data. The specific statistical test used is reported in each figure caption. Mean differences with *p* value < 0.05 were considered statistically significant.

## Conflict of Interest

R.D.K. is a co‐founder of AIM Biotech, a company that markets microfluidic technologies, and receives research support from Amgen, Abbvie, Boehringer‐Ingelheim, GSK, Novartis, Roche, Takeda, Eisai, EMD Serono, and Visterra.

## Supporting information

Supporting Information

## Data Availability

Data and metadata associated with this publication is accessible here: https://fairdomhub.org/studies/1246.
