# Supplementary material for: Personalized Vascularized Models of Breast Cancer Desmoplasia Reveal Biomechanical Determinants of Drug Delivery to the Tumor
Source: Adv Sci (Weinh). 2024 Jul 23;11(38):2402757. doi: 10.1002/advs.202402757 (PMC11481247; doi:10.1002/advs.202402757)
Supplement: Supplementary file 1 — Supporting Information [file ADVS-11-2402757-s001.pdf]

ADVANCED SCIENCE
Open Access

Supporting Information

for *Adv. Sci.*, DOI 10.1002/advs.202402757

Personalized Vascularized Models of Breast Cancer Desmoplasia Reveal Biomechanical Determinants of Drug Delivery to the Tumor

Giovanni S. Offeddu, Elena Cambria, Sarah E. Shelton, Kristina Haase, Zhengpeng Wan, Luca Possenti, Huu Tuan Nguyen, Mark R. Gillrie, Dean Hickman, Charles G. Knutson and Roger D. Kamm**

Supporting Information

Personalized Vascularized Models of Breast Cancer Desmoplasia Reveal Biomechanical Determinants of Drug Delivery to the Tumor

Giovanni S. Offeddu#, Elena Cambria#, Sarah E. Shelton, Kristina Haase, Zhengpeng Wan, Luca Possenti, Huu Tuan Nguyen, Mark R. Gillrie, Dean Hickman, Charles G. Knutson, and Roger D. Kamm**

# These authors contributed equally to this work.
* Corresponding authors

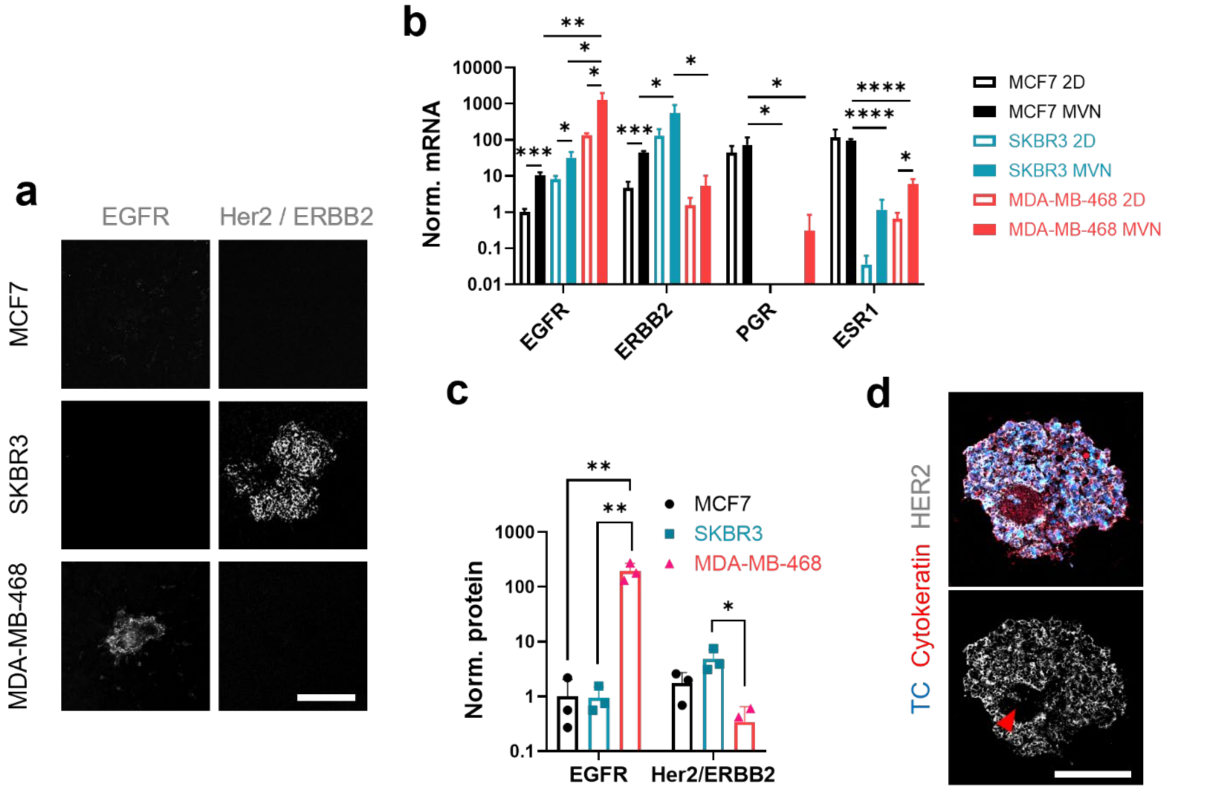

Figure S1. Differential gene and protein expression of target receptors in tumoroid MVNs. (a) Expression of target receptors in tumoroids in MVN devices confirmed by immunofluorescence. The scale bar is 500 µm. (b) Gene expression of target receptors in TCs cultured in 2D or collected from MVN devices; n = 3. Missing bars indicate a non-detectable gene expression. (c) Protein expression of target receptors in 1 mm biopsies centered at the tumoroids in the MVN devices. (d) Projected confocal image of an SKBR3 tumoroid cryosection showing a necrotic core (arrow) where target expression is lost. The scale bar is 500 µm. Significance assessed by one-way ANOVA after confirming normal distribution of the data; in (b), significance is shown only between 2D and MVNs for each TC type, and between TC types in MVN; $p < 0.05$ *, $p < 0.01$ **, $p < 0.001$ ***, $p < 0.0001$ ****.

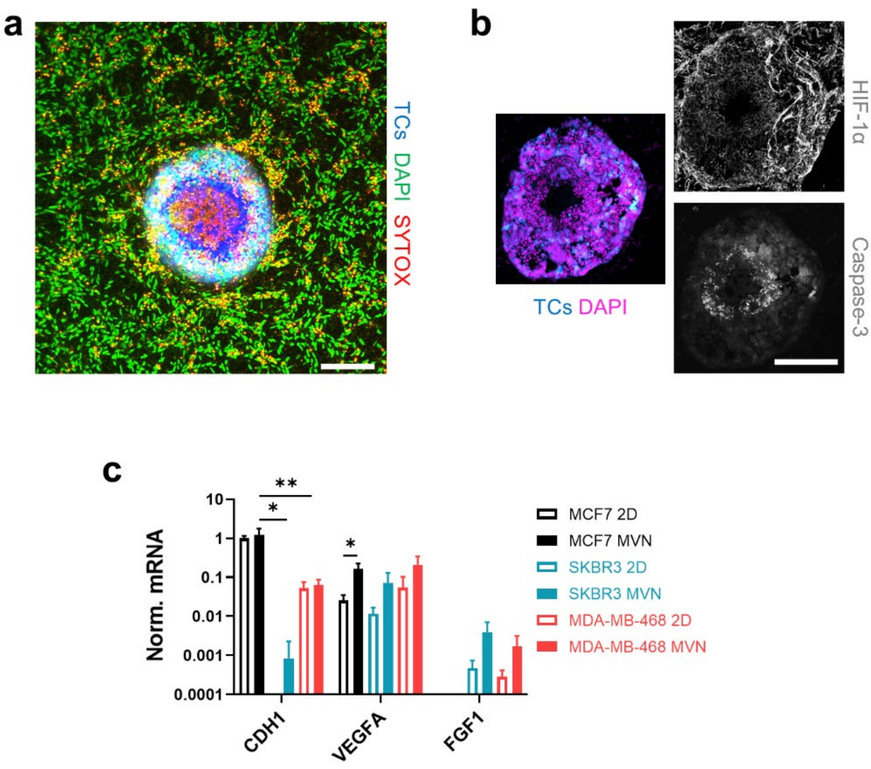

Figure S2. Hypoxia-induced necrosis and gene expression in tumoroid MVNs (a) Projected confocal microscopy image showing a dead core (SYTOX signal) in an MCF7 tumoroid in MVN devices. The scale bar is 200 µm. (b) Confocal image of MCF7 tumoroid cryosection showing elevated HIF-1α. The scale bar is 200 µm. (c) Gene expression in cancer cell lines cultured in 2D or collected from MVN devices; n = 3. Missing bars indicate a non-detectable gene expression. Significance assessed by one-way ANOVA; $p < 0.05$ *, $p < 0.01$ **.

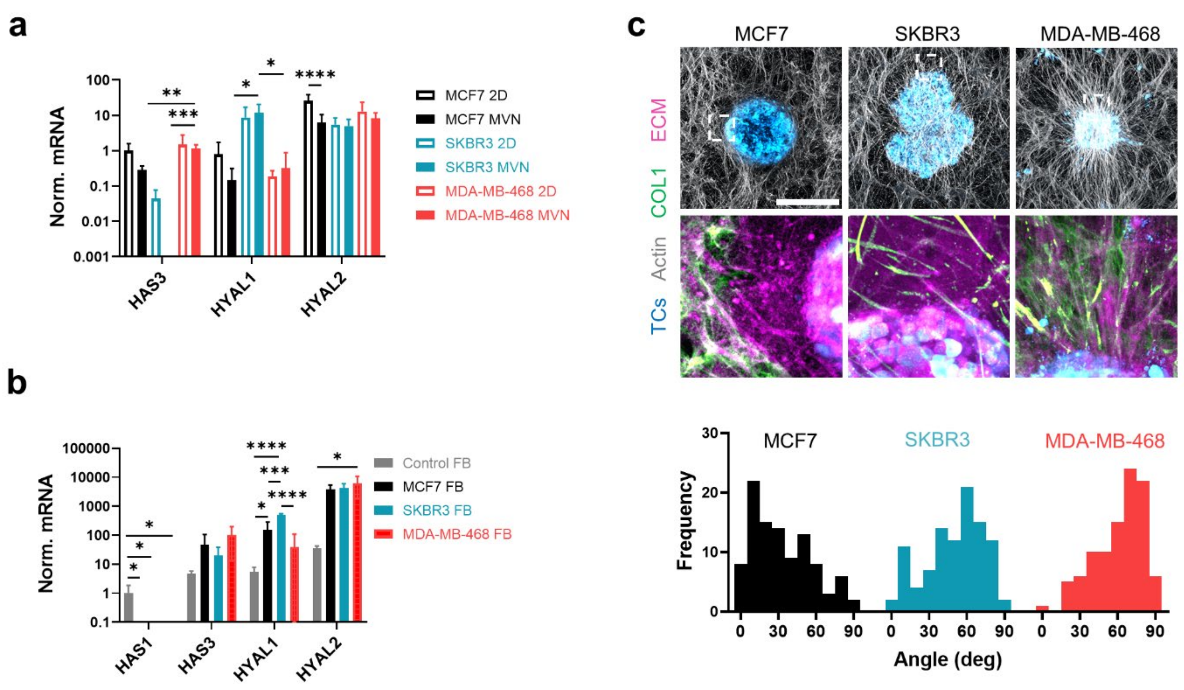

Figure S3. Expression of matrix-associated genes and proteins in tumoroid MVNs. (a) Gene expression in cancer cell lines cultured in 2D or collected from MVN devices; n = 3. Missing bars indicate a non-detectable gene expression. (b) Gene expression in FBs from tumoroid or control MVNs; n = 3. Missing bars indicate a non-detectable gene expression. (c) Confocal images of collagen I in the tumoroid MVNs (top, scale bar = 500 µm), and distributions of the directions of collagen I streaks (bottom); n = 100. Significance assessed by one-way ANOVA; p < 0.05 *, p< 0.01 **, p < 0.001 ***, p < 0.0001 ****.

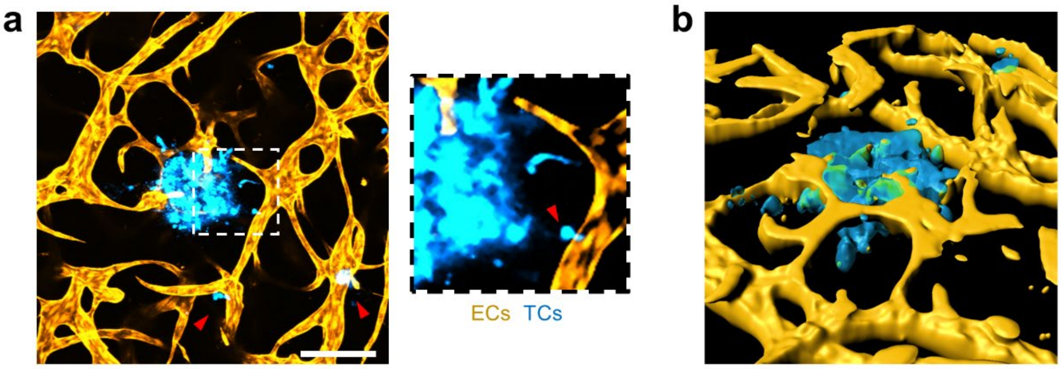

Figure S4. Vascularization of MDA-MB-468 tumoroids. (a) Confocal image of vascularized MDA-MB-468 tumoroid (scale bar = 200 µm) and (b) its 3D reconstruction using the Imaris software showing vessel penetration into the tumoroid mass. The arrows in (a) indicate clusters of invasive TCs. The inset shows a TC intravasating into the MVNs.

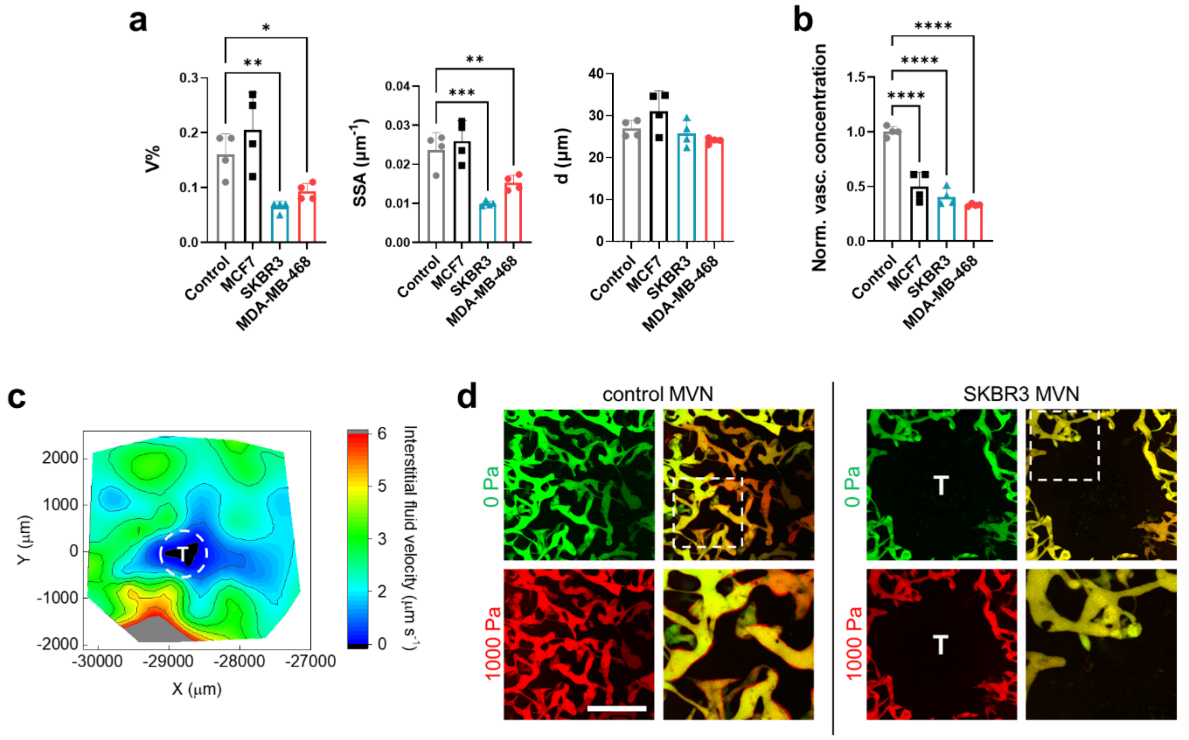

Figure S5. Morphology, perfusability, and evidence of interstitial fluid pressure in tumoroid MVNs. (a) Morphological characterization of control and tumoroid MVNs in terms of volume fraction, *V%*, specific surface area, *SSA*, and vessel diameter, *d*, and (b) normalized dextran concentration in those MVNs, as measured by proxy of fluorescence intensity. (c) Interstitial fluid velocity in the vicinity of an MDA-MB-468 tumoroid in the MVN device, as measured by fluorescence tracking. (d) Fluorescence microscopy images of control and SKBR3 MVNs perfused with dextran as a function of intravascular pressure (left, 0 Pa = green, 1000 Pa = red). Merged images (right) show vessel expansion due to intravascular pressure, evidenced by a red outline. Expansion of the vessels can be seen in pressurized control MVNs, but not in tumoroid MVNs. The scale bar is 500 µm. Significance assessed by one-way ANOVA; $p < 0.5$ *, $p < 0.01$ **, $p < 0.001$ ***, $p < 0.0001$ ****.

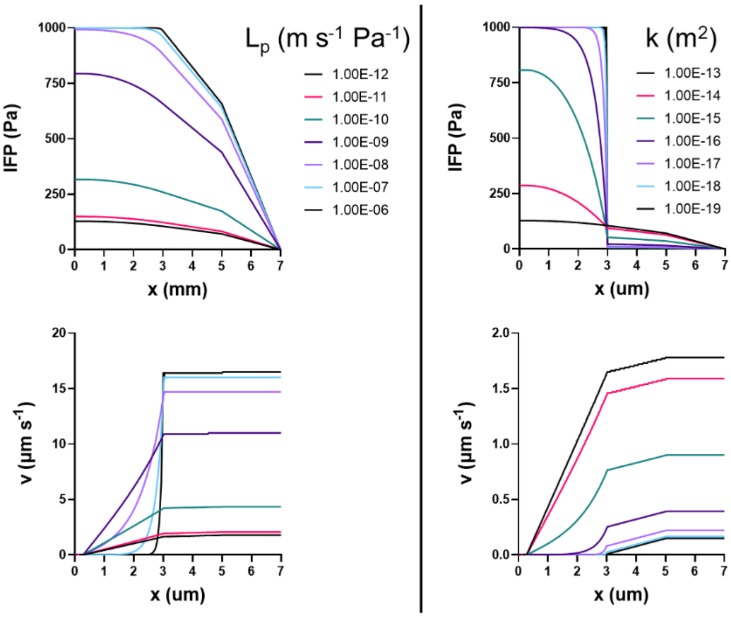

Figure S6. Computational 1D model of interstitial fluid pressure and velocity in tumoroid MVNs. Computational model output of interstitial fluid pressure (top) and interstitial fluid velocity (bottom) as a function of distance from the tumoroids and changes in vascular hydraulic conductivity (left) or matrix permeability (right).

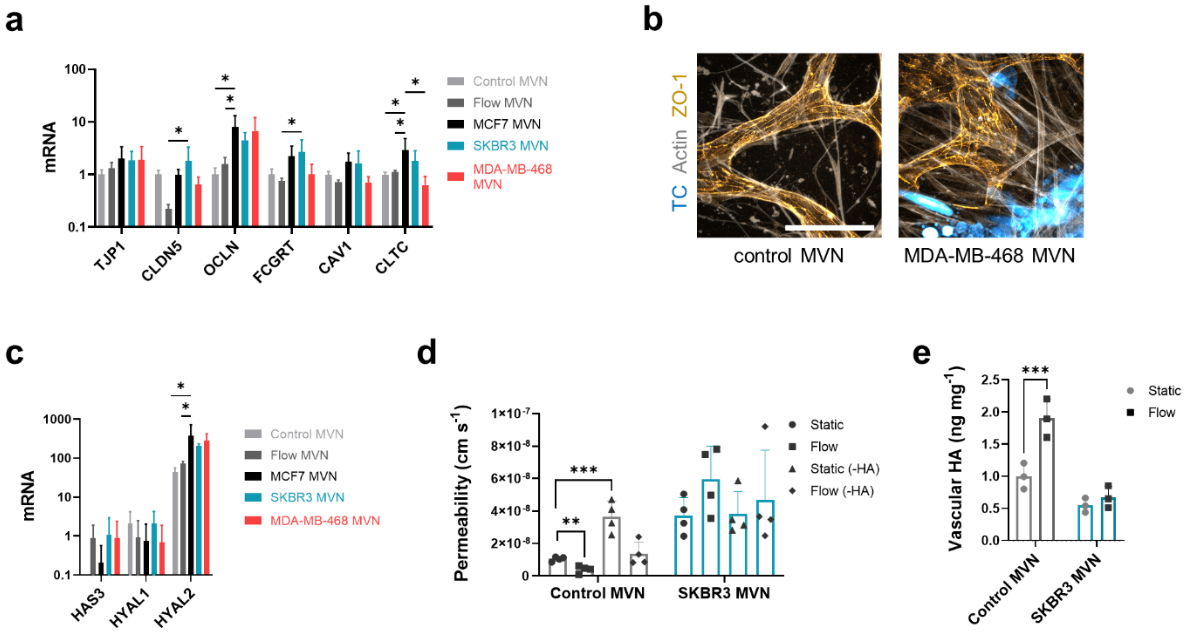

Figure S7. Expression of vasculature-associated genes and proteins in tumoroid MVNs and effect of fluid flow on vascular barrier function. (a) Expression of genes associated with vascular transport in ECs from control or tumoroid MVNs; n = 3. (b) Confocal microscopy image of tight junctions in MVNs. The scale bar is 100 µm. (c) Expression of genes associated with HA production in ECs from control or tumoroid MVNs; n =3. (d) MVN permeability as a function of flow condition and HA degradation by HA-ase in control and SKBR3 MVNs. (e) Vascular HA concentration in control and SKBR3 MVNs cultured statically or under continuous vascular perfusion for 48 hours. Significance assessed by one-way ANOVA; $p < 0.5$ *, $p < 0.01$ **, $p < 0.001$ ***, $p < 0.0001$ ****.

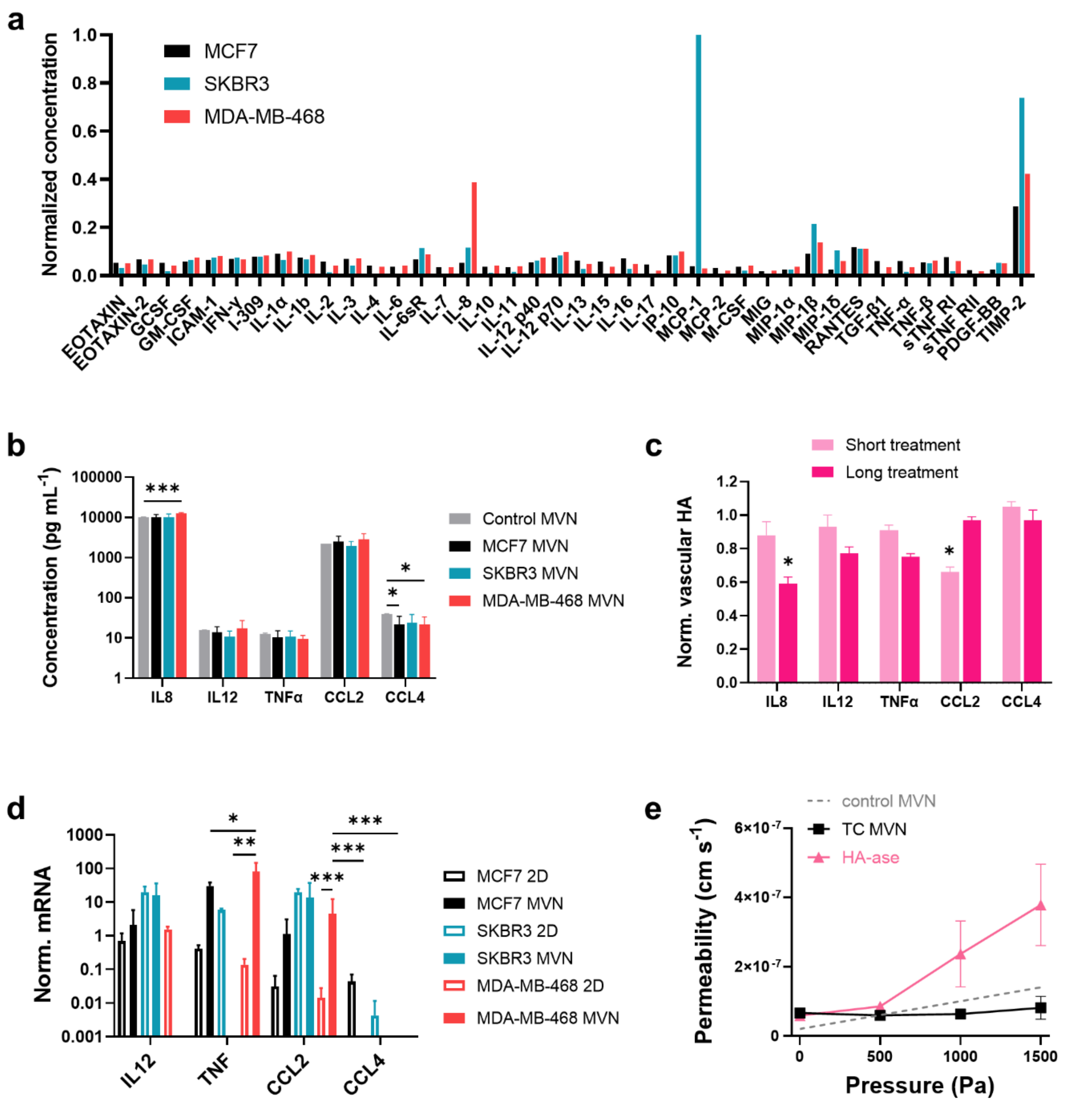

Figure S8. Expression of pro-inflammatory genes and proteins and effect on vascular barrier function. (a) Pro-inflammatory cytokine array in TC-conditioned Vasculife medium. (b) Cytokine concentration in supernatant collected from control and tumoroid MVN devices; n = 3. (c) Quantification of vascular HA by confocal microscopy, normalized to actin signal; n = 3. (d) Expression of genes associated with cytokine production in TCs cultured in 2D or collected from MVN devices; n =3. Missing bars indicate a non-detectable gene expression. (e) Effective permeability of MDA-MB-468 MVNs after intervention with HA-ase; n = 3. Significance assessed by one-way ANOVA; $p < 0.5$ *, $p < 0.01$ **, $p < 0.001$ ***, $p < 0.0001$ ****.

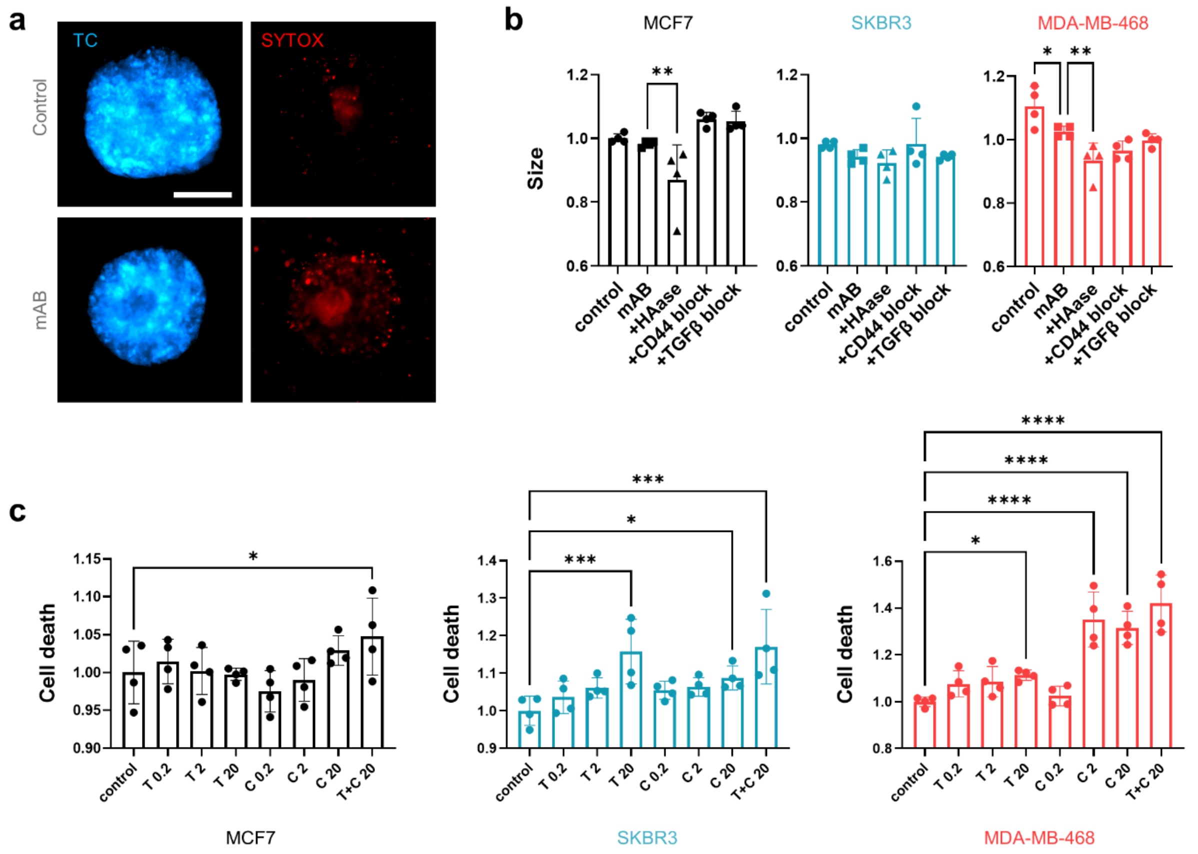

Figure S9. Cell death and size of tumoroids after interventions and drug treatment. (a) Confocal microscopy image of cell death signal in an MCF7 tumoroid in the MVN device after treatment with trastuzumab and cetuximab ("mAB"). (b) Changes in size of cancer cell line tumoroids after combination mAB treatment with interventions targeting stromal HA. (c) Normalized cell death signal in cancer cell line tumoroids in well-plates treated with different concentrations of trastuzumab ("T"), cetuximab ("C"), or both ("T+C"). The concentrations are in μg mL^{-1}. Significance assessed by one-way ANOVA; $p < 0.5$ *, $p < 0.001$ ***, $p < 0.0001$ ****.

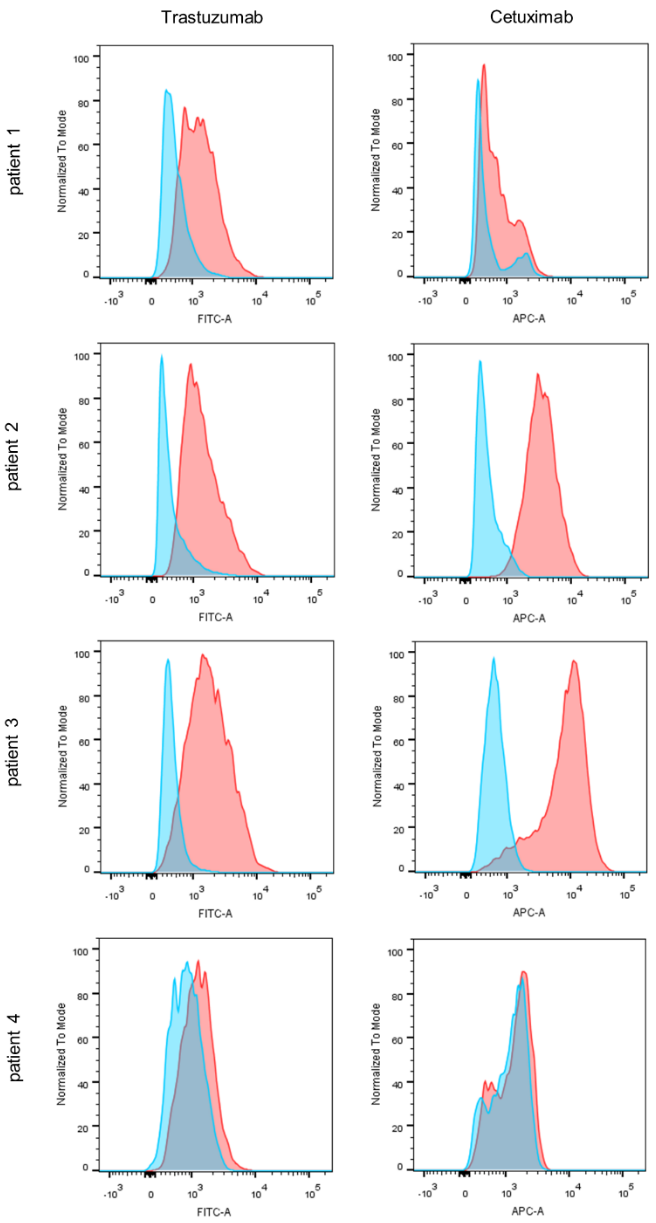

Figure S10. Differential expression of target receptors in patient-derived breast TCs. Expression of HER2/ERBB2 (trastuzumab) and EGFR (cetuximab) in patient-derived breast TCs, as measured by flow cytometry analysis.

Table S1. Values of the parameters used for the computational model analysis.

Variable	Symbol	Unit	Value
Media viscosity	μ	Pa s	10^{-3}
Media density	ρ	kg m^{-3}	10^{3}
Surface to volume ratio	SV_A	m^{-1}	0
	SV_B	m^{-1}	15213
	SV_C	m^{-1}	4000
	SV_D	m^{-1}	0
Hydraulic conductivity of endothelium	$L_{p,A}$	m s^{-1} Pa^{-1}	/
	$L_{p,B}$	m s^{-1} Pa^{-1}	10^{-12} - 10^{-6}
	$L_{p,C}$	m s^{-1} Pa^{-1}	10^{-12}
	$L_{p,D}$	m s^{-1} Pa^{-1}	/
Hydraulic conductivity of monolayer	$L_{p,M}$	m s^{-1} Pa^{-1}	10^{-10}
Permeability of the gel	k_A	m^2	10^{-21}
	k_B	m^2	10^{-19} - 10^{-13}
	k_C	m^2	10^{-13}
(geometrical factor included)	k_D	m^2	0.5 *10^{-13}
Vascular pressure	p_v	Pa	1000
